# SWISS-MODEL: homology modelling of protein structures and complexes

**DOI:** 10.1093/nar/gky427

**Published:** 2018-05-21

**Authors:** Andrew Waterhouse, Martino Bertoni, Stefan Bienert, Gabriel Studer, Gerardo Tauriello, Rafal Gumienny, Florian T Heer, Tjaart A P de Beer, Christine Rempfer, Lorenza Bordoli, Rosalba Lepore, Torsten Schwede

**Affiliations:** 1 Biozentrum, University of Basel, Klingelbergstrasse 50–70, CH-4056 Basel, Switzerland; 2 SIB Swiss Institute of Bioinformatics, Biozentrum, University of Basel, Klingelbergstrasse 50–70, CH-4056 Basel, Switzerland

## Abstract

Homology modelling has matured into an important technique in structural biology, significantly contributing to narrowing the gap between known protein sequences and experimentally determined structures. Fully automated workflows and servers simplify and streamline the homology modelling process, also allowing users without a specific computational expertise to generate reliable protein models and have easy access to modelling results, their visualization and interpretation. Here, we present an update to the SWISS-MODEL server, which pioneered the field of automated modelling 25 years ago and been continuously further developed. Recently, its functionality has been extended to the modelling of homo- and heteromeric complexes. Starting from the amino acid sequences of the interacting proteins, both the stoichiometry and the overall structure of the complex are inferred by homology modelling. Other major improvements include the implementation of a new modelling engine, ProMod3 and the introduction a new local model quality estimation method, QMEANDisCo. SWISS-MODEL is freely available at https://swissmodel.expasy.org.

## INTRODUCTION

Three-dimensional structures of proteins provide valuable insights into their function on a molecular level and inform a broad spectrum of applications in life science research. Often, complexes of proteins are central to many cellular processes. A detailed description of their interactions and the overall quaternary structure is essential for a comprehensive understanding of biological systems, how protein complexes and networks operate and how we can modulate them (1,2). Given their biological relevance, it is not surprising that the number of large complexes deposited per year in the Protein Data Bank (PDB) is growing rapidly (3). A significant contribution to this trend originates from the continuous progress of structure determination technologies, including recent developments of Electron Microscopy (EM) based methods, which are particularly suited for large macromolecular assemblies (4). Still, compared to high-throughput methods for screening protein-protein interactions (i.e. yeast two-hybrid, affinity purification, phage-display etc.), the rate at which novel complex structures are determined experimentally is considerably lower. This uneven growth calls for computational methods to fill the gap.

Several approaches have been developed to address the computational prediction of protein-protein interactions (5). Co-evolution methods, based on correlated amino acid mutations in deep multiple sequence alignments (MSA), are efficiently used to identify interacting proteins based on sequence information alone (6,7). When the 3D structures of the binding partners are available, or can be reliably modelled, docking methods can be used to obtain a three-dimensional model of the complex based on geometric and physicochemical complementarity of the interacting molecules (8–11). Efficiently handling protein flexibility is still one of the major challenges in the development of effective docking simulation software; hence these methods are generally more accurate when little or no conformational change is required for binding. According to the community-wide experiment CAPRI (Critical Assessment of PRedicted Interactions (12)), considerable progress has been made in the field with the development of hybrid modelling strategies, that are able to incorporate available experimental information on the interaction (i.e. crosslinks, NMR, SAXS etc.) as constraints in the simulation of the docking process (13–15). Results from latest assessments show that significantly improved quality of models is obtained when multi-chain template information is available and used for modelling (16).

With more experimentally determined structures of protein complexes becoming available, it has been observed that interacting interfaces are often conserved among homologous complexes (17) and that templates are available for most of the known protein-protein interactions (18). These observations provided the rationale for comparative, or homology modelling, of protein complexes. Similar to comparative modelling of monomeric proteins, the information of a protein's quaternary structure is transferred by homology to another one, and a model of the complex is obtained based on the structures of the interacting homologs, i.e. interologs, as templates (19–21). The approach can be scaled to entire genomes and applied to binary as well as to higher-order protein assemblies (17,18,22,23). As highlighted by the introduction of the first assessment of protein assemblies in the recent CASP XII experiment (24), comparative modelling of protein complexes is receiving much attention and is expected to play a relevant role in the elucidation of the protein quaternary structure space.

SWISS-MODEL https://swissmodel.expasy.org was the first fully automated protein homology modelling server and has been continuously improved during the last 25 years (25–30). Its modelling functionality has been recently extended to include the modelling of homo- and heteromeric complexes, given the amino acid sequences of the interacting partners as starting point. Other recently introduced features include the development of a new modelling engine, ProMod3, with increased accuracy of the produced models, and an improved local model quality estimation method (QMEANDisCo) based on a novel version of QMEAN (31).

SWISS-MODEL currently generates ∼3000 models a day (∼2 models per minute), up from ∼1500 models a day in 2014 (30), making it one of the most widely used structure modelling servers worldwide. Its performance is continuously evaluated and compared with other state-of-the art servers in the field. To this aim, we are actively participating to the CAMEO project (Continuous Automated Model Evaluation, https://cameo3d.org) (32), a fully automated blind prediction assessment based on weekly pre-release of sequences from the PDB (33), allowing us to constantly monitor and improve the performance of the server.

## MATERIALS AND METHODS

### The modelling workflow

In comparative modelling, a 3D protein model of a target sequence is generated by extrapolating experimental information from an evolutionary related protein structure that serves as a template. In SWISS-MODEL, the default modelling workflow consists of the following main steps:

1. **Input data**: The target protein can be provided as amino acid sequence, either in FASTA, Clustal format or as a plain text. Alternatively, a UniProtKB accession code (34) can be specified. If the target protein is heteromeric, i.e. it consists of different protein chains as subunits, amino acid sequences or UniProtKB accession codes must be specified for each subunit.
2. **Template search**: Data provided in step 1 serve as a query to search for evolutionary related protein structures against the SWISS-MODEL template library SMTL (30). SWISS-MODEL performs this task by using two database search methods: BLAST (35,36), which is fast and sufficiently accurate for closely related templates, and HHblits (37), which adds sensitivity in case of remote homology.
3. **Template selection**: When the template search is complete, templates are ranked according to expected quality of the resulting models, as estimated by Global Model Quality Estimate (GMQE) (30) and Quaternary Structure Quality Estimate (QSQE) (23). Top-ranked templates and alignments are compared to verify whether they represent alternative conformational states or cover different regions of the target protein. In this case, multiple templates are selected automatically and different models are built accordingly. To provide the user with the option to use alternative templates than those selected automatically, all templates are shown in a tabular form with a descriptive set of features. In addition, interactive graphical views facilitate the analysis and comparison of available templates in terms of their three-dimensional structures, sequence similarity and quaternary structure features.
4. **Model building**: For each selected template, a 3D protein model is automatically generated by first transferring conserved atom coordinates as defined by the target-template alignment. Residue coordinates corresponding to insertions/deletions in the alignment are generated by loop modelling and a full-atom protein model is obtained by constructing the non-conserved amino acid side chains. SWISS-MODEL relies on the OpenStructure computational structural biology framework (38) and the ProMod3 modelling engine to perform this step. For more detailed information on model building we refer to a dedicated section in Results.
5. **Model quality estimation**: To quantify modelling errors and give estimates on expected model accuracy, SWISS-MODEL relies on the QMEAN scoring function (31). QMEAN uses statistical potentials of mean force to generate global and per residue quality estimates. The local quality estimates are enhanced by pairwise distance constraints that represent ensemble information from all template structures found. For more information on quality estimation we refer to a dedicated section in Results.

SWISS-MODEL allows for further customization of steps 1 and 3. Expert users can directly upload custom target-template sequence alignments, template structures or DeepView project files (26) in separate input forms.

### The SWISS-MODEL template library

The SWISS-MODEL Template Library (SMTL), available at https://swissmodel.expasy.org/templates/, is a curated template library, which is updated on a weekly basis according to the new PDB release (33). Every deposited PDB structure is automatically processed, annotated and indexed to support efficient querying of high quality structural data. SMTL entries are organized by quaternary structure assemblies, according to the ‘Biological Assembly’ annotation specified in the PDB. Biologically relevant ligands are annotated accordingly, as described in (30), and the annotation is then used by the modelling engine to determine whether a ligand is considered for inclusion into the final model. As of January 2018, the SMTL contains coordinates for a total of 92 474 unique protein sequences, mapping to 219 350 biological units, annotated as follows: 113 639 monomers, 71 555 homo-oligomers and 34 156 hetero-oligomers.

### Integration with the SWISS-MODEL repository and cross-links to other services

The SWISS-MODEL Repository (39) (SMR, https://swissmodel.expasy.org/repository) is a database of automatically generated homology models for relevant model organisms and experimental structure information for all sequences in UniProtKB (34). Whenever a UniProtKB sequence is submitted to SWISS-MODEL, the generated model is automatically deposited into the SMR along with all data used to generate the model. Currently, the SMR contains 1 067 355 models from SWISS-MODEL and 129 416 structures from PDB with mapping to UniProtKB.

To facilitate exploration of available information on a given target protein, SWISS-MODEL provides cross-links to various other resources and databases. We include links to the RCSB (33), PDBsum (40), PDBe (41), CATH (42) and SwissDock (43). In addition, we also provide direct access to a specialised server for antibody modelling. The pre-screening of the target sequence has been extended in order to automatically identify whether an immunoglobulin sequence is present in the input. If a matching sequence signal is detected, data can be sent to the Prediction of Immunoglobulin Structure server PIGSPro (44–46) where the model of the antibody is generated according to the canonical structure method (47–49).

### Documentation and technical implementation

An updated version of the documentation is provided to reflect the latest changes of the current SWISS-MODEL release. Tutorial pages and examples have been updated according to latest options and features available. Additionally, a video tutorial with a step-by-step guide on how to generate a model using SWISS-MODEL is available at https://swissmodel.expasy.org/docs/tutorial.

SWISS-MODEL is implemented in Python and Django with Javascript and jQuery for the front-end, and Python, C++ and OpenStructure (38) for the back-end functions. For visualization of protein structures, users can select between two interactive JavaScript/WebGL molecule viewers, PV (https://biasmv.github.io/pv/) and NGL (https://github.com/arose/ngl) (50). ProMod3 was developed using OpenStructure; its core is written in efficient C/C++ and a Python interface is provided for rapid prototyping.

## RESULTS AND DISCUSSIONS

### The ProMod3 modelling engine

The modelling engine is the heart of SWISS-MODEL. It builds an atomistic protein model given a template structure and a target-template sequence alignment. Until recently, the software package ProMod-II (26), using MODELLER (51) as a fall-back, was in use to perform this task. As of June 2016, the newly developed modelling engine ProMod3 is used exclusively. ProMod3 has been designed with the aim of providing rapid and flexible prototyping for future modelling developments in SWISS-MODEL.

Like its predecessors, ProMod3 extracts structural information from an aligned template structure in Cartesian space. Insertions and deletions, as defined by the sequence alignment, are resolved by first searching for viable candidate fragments in a structural database. This is a relevant modification, as ProMod-II mainly relied on *ab-initio* techniques to perform this step. Final candidates are selected using statistical potentials of mean force scoring methods. If no suitable fragments can be found, a conformational space search is performed using Monte Carlo sampling. Non-conserved side-chains are modelled using the 2010 backbone dependent rotamer library from the Dunbrack group (52). The optimal configuration of rotamers is estimated using the graph based TreePack algorithm (53) by minimizing the SCWRL4 energy function (54). As a final step, small structural distortions, unfavourable interactions or clashes introduced during the modelling process, are resolved by energy minimization. ProMod3 uses the OpenMM library (55) to perform the computations and the CHARMM27 force field (56) for parameterization.

**Figure 1.**
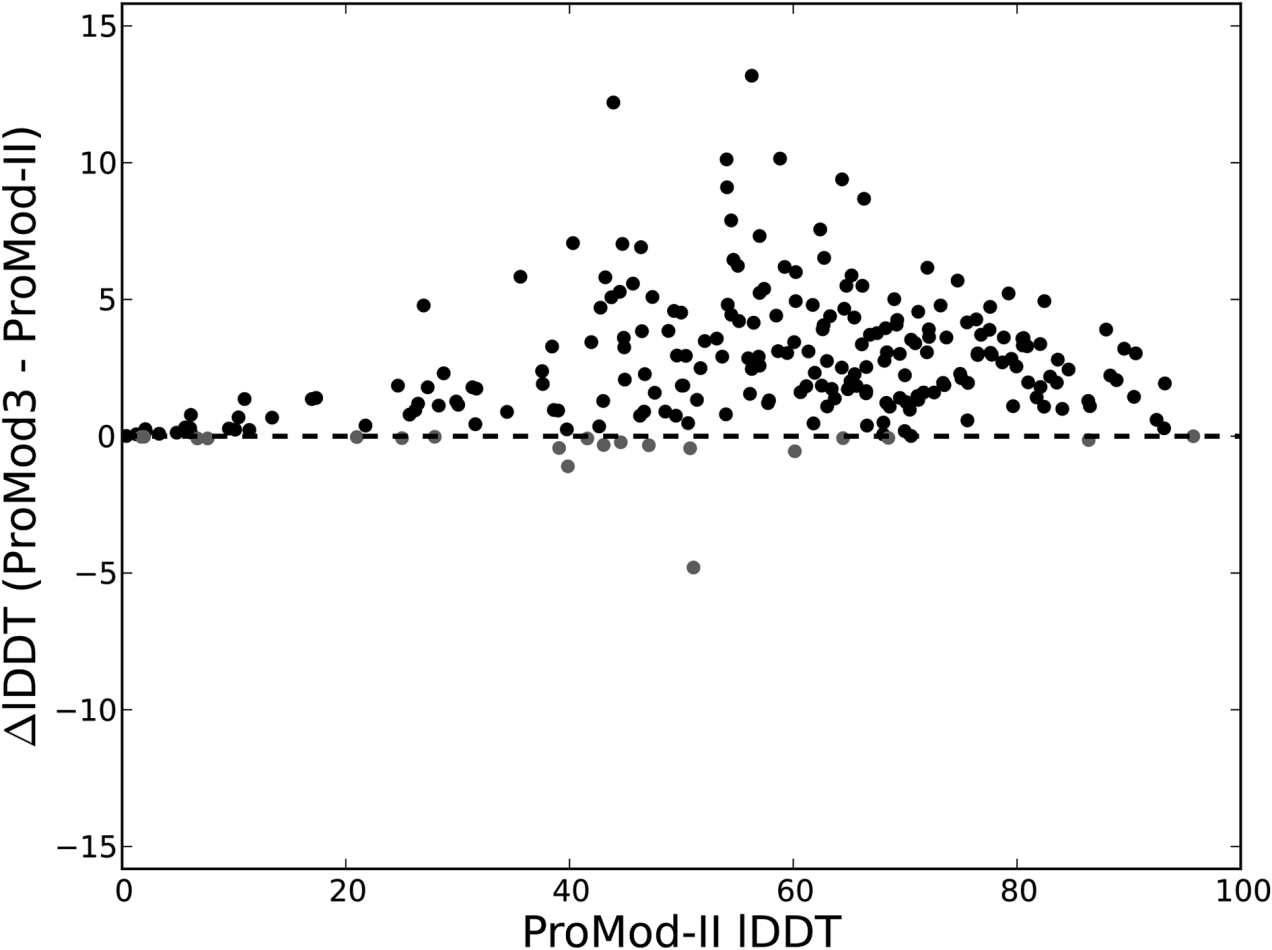
Performance comparison between ProMod-II and ProMod3 modelling engines. Performance is measured on a benchmark dataset of 250 targets collected during the CAMEO time range 20 October 2017–13 January 2018. For each target, the same template and target-template alignment were used as input for both modelling engines. Each data point represents the difference in model accuracy in terms of all-atom IDDT score. ProMod3 shows a statically significant improvement of 2.65 IDDT points on average (*P*-value = 1.1E–43) based on paired *t*-test.

A direct comparison between the previous and updated modelling engines has been performed in the context of the CAMEO experiment using 250 target proteins collected during the time range 20 October 2017–13 January 2018. For each target, a template search has been performed using HHblits against the SMTL at the time of the CAMEO submission. The best template, according to the HHblits *e*-value, and the corresponding target-template sequence alignment served as input for both engines. As shown in Figure 1, models generated with ProMod3 show significantly improved accuracy according to all-atom lDDT (Local Distance Difference Test) score (57), a superposition-free measure of the deviation of interatomic distances between model and native structures. The same also holds for other commonly used model quality metrics, i.e. GDT-HA (Global Distance Test High Accuracy score) (58) and TM-score (Template Modelling score) (59) (Supplementary Figure S1).

### Modelling the protein quaternary structure of homo- and hetero-oligomers

In SWISS-MODEL, we have recently introduced a new approach to model the stoichiometry and overall structure of protein complexes using the sequence of the interacting component as starting points (23). The method is based on a novel description of interface conservation as a function of evolutionary distance. The basic assumption is that biologically relevant interfaces are less free to vary than the rest of the protein surface (60,61). We capture such evolutionary constraints by measuring the ratio between interface and surface residue entropy distribution from multiple sequence alignments (MSA) of homologous proteins as a function of evolutionary distance. We employ this analysis, termed PPI fingerprint, to discriminate biologically relevant interfaces from crystal contacts, and for estimating the accuracy of models. Interface conservation analysis and geometric properties of protein complexes were used to train a supervised machine-learning algorithm, Support Vector Machines (SVM), to identify templates that maximize the estimated interface quality of the resulting model. The predicted interface quality, i.e. quaternary structure quality estimate (QSQE), is a score between 0 and 1 reflecting the expected accuracy of inter-chain contacts in a model based on a given alignment and template. Further details are provided in (23).

### Model quality estimation

SWISS-MODEL provides quality estimates at several stages of the modelling process. Given a template structure and target-template alignment, the GMQE (30) and the QSQE (23) provide estimates of the expected quality of the resulting model at the tertiary and quaternary structure level. These estimates help the user identify optimal templates and are also utilized for the fully automated template selection procedure. Once models have been built, their quality is assessed by the QMEAN scoring function (31). QMEAN employs statistical potentials of mean force to generate quality estimates on a global and local scale. The latest version of QMEAN, QMEANDisCo, further enhances the accuracy of local quality estimates. It assesses the consistency of observed interatomic distances in the model with ensemble information extracted from experimentally determined protein structures that are homologues to the target sequence. To incorporate structural features, GMQE is updated after model building with the QMEAN global score and is then used for the model ranking. To facilitate interpretation of the obtained model quality estimates, the QMEAN global score is transformed to a *Z*-score, indicating whether the model scores as it would be expected from experimentally determined structures of similar size (31).

### Performance comparison with other modelling servers

In order to provide objective assessments of modelling performance, SWISS-MODEL participates in the CAMEO project (https://cameo3d.org) (32). Taking some inspiration from CASP, CAMEO aims to provide a continuous, fully automated, assessment of predictions produced by various modelling servers using a common benchmark dataset of targets. CAMEO target sequences are obtained from the weekly pre-release of new PDB structures and submitted to participating methods at the same point in time. This ensures all servers have access to the same background information, i.e. same structures from PDB or protein sequences in UniProtKB, when running their predictions. Finally, in order to exclude trivial modelling cases, protein sequences exhibiting >85% sequence identity to available PDB structures are not considered in the CAMEO evaluation.

Based on the CAMEO results in the ‘3D Structure Prediction’ category, SWISS-MODEL is consistently ranked among the top-modelling servers for several crucial modelling aspects. Table 1 shows the performance based on a benchmark dataset of 250 targets collected during the CAMEO time range 20 October 2017–13 January 2018. Full data on performance are provided as supplementary materials (Supplementary Tables S1–S7). Notably, SWISS-MODEL has the lowest response time to generate models and excels at model quality for binding sites (IDDT-BS), for high-quality models (lDDT-easy) and for quaternary structure prediction (QS-score). SWISS-MODEL is optimized for comparative protein modelling cases, where high-quality models can be generated and used in a variety of practical research applications (62). For difficult remote homology or *de novo* modelling targets, other methods perform better in the CAMEO assessment (63–65). It is worth mentioning that among the participating servers, only SWISS-MODEL and Robetta provided results for oligomeric targets. Therefore quaternary structure predictions were assessed on a common subset of oligomeric proteins where both methods returned a model, for a total of 32 targets. Finally, based on the assessment of model confidence, SWISS-MODEL significantly outperforms other modelling servers in providing accurate local confidence estimates of the returned models.

**Table 1.** Performance comparison in the context of the CAMEO continuous evaluation platform

Server	Response time (hh:mm:ss) (*N* = 168)	lDDT total (*N* = 168)	lDDT easy (*N* = 37)	lDDT medium (*N* = 90)	lDDT hard (*N* = 41)	lDDT BS (*N* = 69)	QS-Score (*N* = 32)	Model confidence (*N* = 168)
SWISS-MODEL	00:15:48	66.22	86.01	69.71	40.67	70.88	63.95	0.85
HHpredB	01:16:15*	65.95	82.10*	69.68	43.18	71.47	-	0.79*
NaiveBLAST	01:20:27*	58.93*	82.86*	64.20*	25.76*	63.88*	-	0.68*
PRIMO	02:12:08*	60.26*	84.51*	65.07*	27.82*	67.30*	-	0.67*
SPARKS-X	02:35:21*	63.14*	80.06*	65.57*	42.53	67.76*	-	0.54*
RaptorX	06:28:57*	69.15*	83.35*	72.10*	49.88*	68.85	-	0.65*
IntFOLD4-TS	32:47:59*	68.41*	83.76*	70.88	49.11*	71.65	-	0.84
Robetta	37:00:07*	71.60*	85.17	74.00*	54.08*	67.48*	60.20	0.81*

Performance is measured based on a benchmark dataset of 250 targets collected during the CAMEO time range 20 October 2017–13 January 2018. Results from SWISS-MODEL and seven other modelling servers were collected from CAMEO and the performance evaluated on a common subset of targets where all compared servers returned a model. Each column indicates average performance values in terms of Response Time, model accuracy (IDDT, QS-score) and self-assessment of model quality (Model Confidence). lDDT evaluation has further been split according to CAMEOs definition of target difficulty; per column subset sizes are shown in brackets. Asterisks indicate a statistically significant difference (*P*-value < 0.05) compared to SWISS-MODEL based on paired *t*-test.

### Case study: Modelling the Ferredoxin/Ferredoxin-NADP(+) Reductase complex

To illustrate the new features of SWISS-MODEL, we describe here the modelling of the hetero-dimeric complex formed by Ferredoxin-NADP(+) Reductase (FNR) and its physiological electron donor Ferredoxin (Fd). In higher plants, these proteins are part of the electron transport chain of thylakoid membranes where they catalyse the last step of NADP+ reduction. In non-photosynthetic tissues, i.e. roots, the reaction operates in the opposite direction and is mediated by the tissue specific isoforms of the enzymes (66). Crystal structures of the leaf electron transfer complex FNR:Fd have been reported from *Zea mays*, providing structural details of the protein–protein interactions involved in electron transfer during light dependent reactions of photosynthesis (67). Only recently, a three-dimensional structure of the FNR:Fd complex formed by the root isotypes has been determined (68). To illustrate the modelling of the root FNR:Fd complex, the native structure has been removed from the SMTL and is used only for validation and visualization purposes.

**Figure 2.**
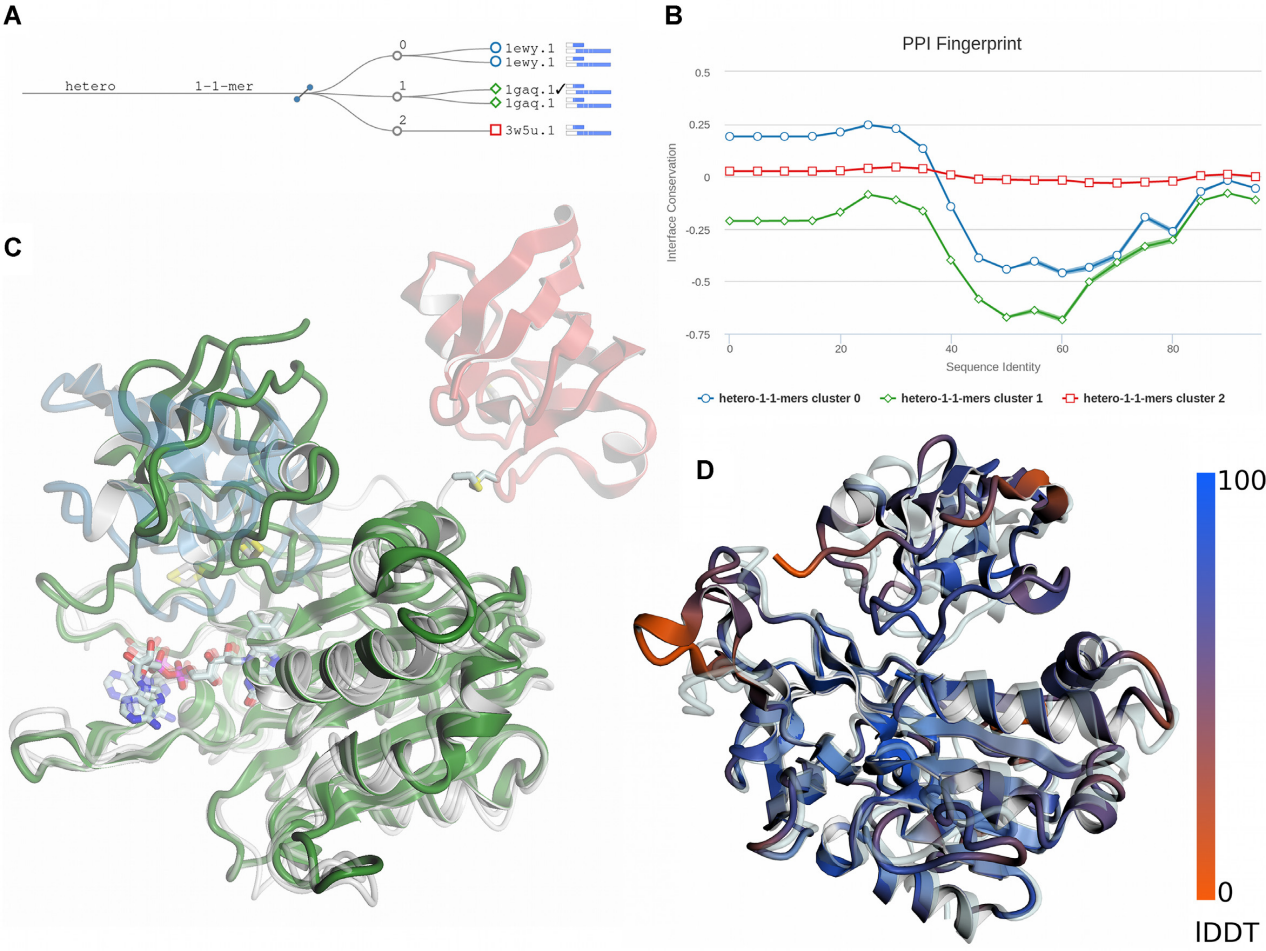
Modelling example of the Ferrodoxin/Ferredoxin-NADP(+) Reductase hetero dimeric complex. (**A**) Decision tree of templates clustered according to their quaternary structure features: oligomeric state, stoichiometry, topology and interface similarity. Three different clusters are formed based on interface similarity between templates. (**B**) PPI fingerprint analysis of available template structures. The ratio between interface and surface residue entropy (interface conservation, y-axis) is reported as a function of evolutionary distance (sequence identity, x-axis). Templates corresponding to SMTL ID: 1ewy.1 (in blue) and SMTL ID: 1gaq.1 (in green) show the typical conservation pattern observed for biologically relevant interfaces, with stronger conservation signal in the sequence identity range between 40 and 60%. Considering also remote homologs (below 40% sequence identity), only the interface in template SMTL ID: 1gaq.1 is deemed as conserved (interface/surface conservation ratio below zero). Template corresponding to SMTL ID: 3w5u.1 (in red) displays an interface/surface conservation ratio close to zero, as observed in crystal contacts/artefacts. (**C**) Structure superposition of available templates. Each template is coloured according to same colouring scheme of Figure 2A and B. Templates corresponding to SMTL ID: 1ewy.1 (in blue) and 1gaq.1 (in green) show similar arrangement of FNR and Fd in the complex. Template SMTL ID: 3w5u.1 (red) shows a different localization of the Fd moiety. Cross-linked cysteines are shown in sticks. (**D**) Structure superposition between model and native structure of the root FNR:Fd complex. The model is coloured according to its local quality using a colour gradient from blue (high quality) to red (low quality) as measured by all-atom IDDT score. The native structure of the complex is shown in light gray.

The amino acid sequences of root FNR (UniProtKB: B4G043) and root Fd (UniProtKB: P27788) from *Z. mays* were submitted to SWISS-MODEL. Results of the template search are shown in Figure 2A, where templates are clustered and displayed in a decision tree according to their quaternary structure features: oligomeric state, stoichiometry, topology and interface similarity. Each leaf of the tree corresponds to a template and target-template alignment (based on HHblits, BLAST or both); templates are labelled with their SMTL ID; bars indicate sequence identity and coverage to the target (darker shades of blue indicate higher sequence identity). Three homologous template complexes could be identified, with similar coverage and identity to the target sequences: FNR sequence coverage between 75 and 78%, sequence identity between 50 and 52%; Fd sequence coverage 63–64% and sequence identity between 66 and 70%. Based on clustering results, available templates have the same oligomeric state, stoichiometry and topology. In terms of structural interface similarity, on the other hand, they form three different clusters. As shown in the PPI fingerprint plot (Figure 2B), two templates (SMTL ID: 1gaq.1 (67) and SMTL ID: 1ewy.1 (69)) display a similar interface conservation pattern, typical to that observed for biologically relevant interfaces (23). Instead, 3w5u.1 shows a different PPI fingerprint curve, with conservation score constantly close to zero, as typically observed for crystallization artefacts (23,70). Notably, the quality of the model interface based on this template is expected to be very low (QSQE = 0.13). Indeed, after inspecting the structure and the corresponding study (71), we could confirm that the interface observed in the 3w5u.1 biounit is the result of a cross-link experiment and, as it can be appreciated in Figure 2C, does not correspond to the biologically functional interface. A stronger conservation signal is visible for template SMTL ID: 1gaq.1 (green line in Figure 2B), which according to both tertiary and quaternary structure quality estimates (GMQE = 0.62; QSQE = 0.54) is considered the best template among the available options; hence it is selected for modelling. The resulting model is shown in Figure 2D, where it has been superimposed onto the experimental structure of the complex (PDB ID: 5H5J (68), shown in light gray). Results of local quality assessment can be visualized onto the model where the colour gradient, from blue to red, indicates high to low quality as measured according to all-atom IDDT score. As it can be observed, three-dimensional structures of both FNR and Fd are modelled with good accuracy (Cα-RMSD: FNR = 2.8Å; Fd = 1.6 Å; IDDT: FNR = 77, Fd = 74 Å). Only small regions, mostly found on surface loops or terminal tails, show lower quality compared to the rest of the protein structures. The relative arrangement of the two proteins in the modelled complex is similar to that observed in the native structure, but the Fd subunit has a different orientation. The QS-score, which expresses the fraction of shared interface contacts between model and native complex, is 0.52. This value is higher than what we observe when comparing biologically functional FNR:Fd complexes, i.e. the templates and the native structure of the target protein, where the pairwise QS-scores range from 0.10 to 0.48, probably due to the elusive localization of the Fd moiety in crystals structures (69). Notably, the interface quality of the model, i.e. QS-score = 0.52, agrees very well with its estimated accuracy, i.e. QSQE = 0.54. The same correspondence is also observed between the local model quality estimated by QMEANDisCo and that measured according to all-atom IDDT score (Pearson correlation on the full complex = 0.80; Supplementary Figure S2). Finally, the FNR:Fd complex model was compared to that obtained using our previous modelling engine, ProMod-II. An average improvement of 2.5 IDDT points per chain is obtained with ProMod3. This is consistent with the results of our performance evaluation on a benchmark dataset (Figure 1).

## CONCLUSIONS

Computational structural modelling methods have established themselves as a valuable complement to experimental structural biology efforts towards increasing our understanding of the protein universe and of its properties. In this endeavour, comparative modelling techniques have matured into fully automated pipelines, providing easy access to reliable 3D models and broadening the spectrum of users and applications of protein models. SWISS-MODEL pioneered the field of fully automated comparative modelling servers 25 years ago and it has been continuously developed and improved since then.

With the new version of SWISS-MODEL presented here, we aimed at extending the scope of automated homology modelling to address the modelling of protein assemblies by efficiently using the information on quaternary structures available in the PDB. The success of this approach clearly depends on the availability of homologous complexes that can be used as templates for modelling. As such, ongoing structural biology efforts leading to structures of macromolecular complexes being determined at unprecedented speed are tremendously beneficial for making our approach increasingly applicable and effective. An important aspect is the ability to handle ambiguous or conflicting information present in available structural data, which is crucial for the development of stable and fully automated pipelines. Here, we showed how our PPI fingerprint analysis and model quality estimates could provide additional criteria to improve the automatic identification of templates, which in turn results into more accurate models and a biologically meaningful representation of their oligomeric state. Finally, we introduced an improved modelling engine and increased the precision of model quality estimates, leading to more accurate models and realistic error estimates at the same time.

## Supplementary Material

Supplementary Data
