# Supplementary material for: SWISS-MODEL: homology modelling of protein structures and complexes
Source: Nucleic Acids Res. 2018 May 21;46(Web Server issue):W296–303. doi: 10.1093/nar/gky427 (PMC6030848; doi:10.1093/nar/gky427)
Supplement: Supplementary Data [file gky427_supplemental_files.pdf]

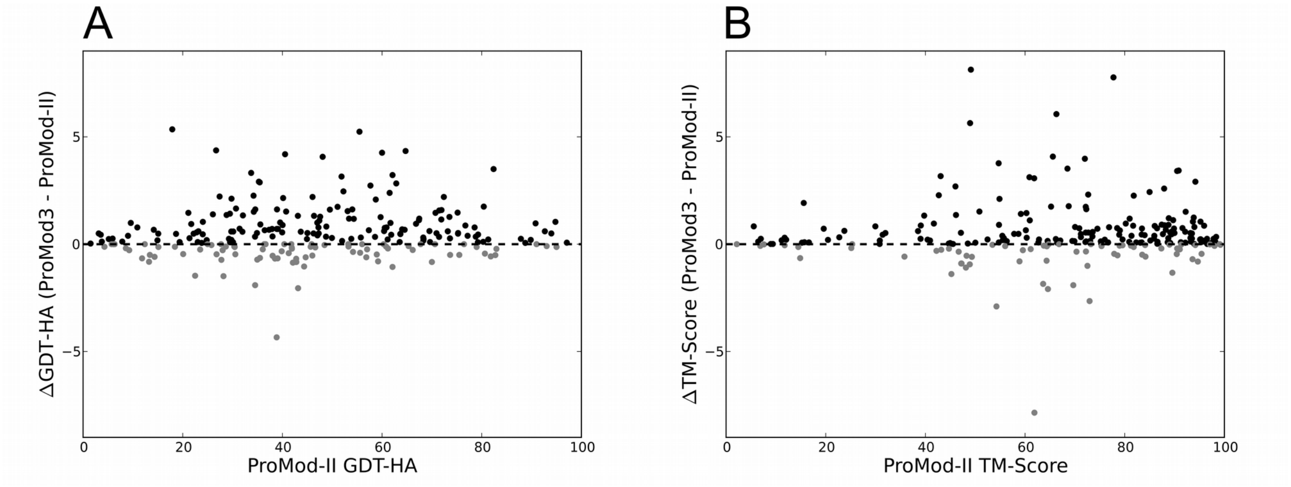

Supplementary Figure 1. Performance comparison between ProMod-II and ProMod3 modelling engines. Performance is measured on a benchmark dataset of 250 targets collected during the CAMEO time range 2017-10-20 - 2018-01-13. For each target, the same template and target-template alignment were used as input for both modelling engines. Every data point represents the difference in model accuracy in terms of (A) GDT-HA and (B) TM-Score. ProMod3 shows an average improvement of 0.54 GDT-HA points and 0.46 TM-Score points (statistically significant difference, P-values: 1.45E-11 and 9.96E-07 based on paired t-test).

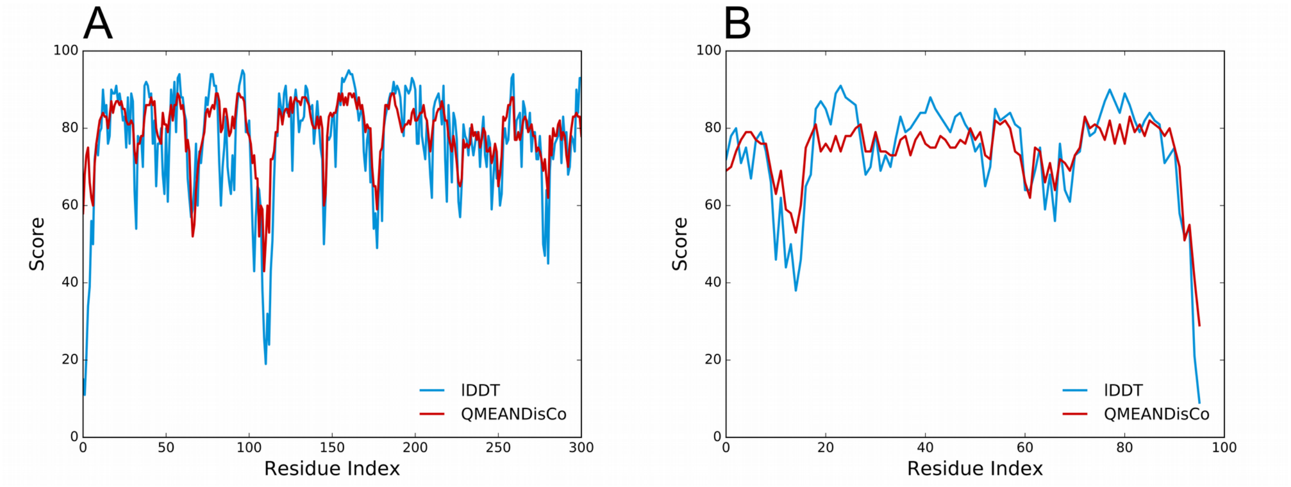

Supplementary Figure 2. Model confidence evaluation of (A) Ferredoxin-NADP+ Reductase and (B) Ferredoxin. Per residue quality estimates from QMEANDisCo are compared to the measured quality by all-atom lDDT scores (Pearson correlation on full complex: 0.80).

Raw data – Response Time

Target	SWISS-MODEL	HHpredB	NaiveBLAST	PRIMO	SPARKS-X	RaptorX	IntFOLD4-TS	Robetta
**2018-01-13_00000090_1**	00:21:17	00:28:01	02:57:12	01:16:40	02:21:17	06:45:46	39:33:23	64:00:01
**2018-01-13_00000073_1**	00:06:49	00:23:46	02:57:12	00:53:25	02:10:49	07:16:32	30:57:54	63:29:46
**2018-01-13_00000068_1**	00:06:32	00:24:17	00:00:00	00:46:56	02:08:33	07:19:03	31:04:36	63:46:17
**2018-01-13_00000066_2**	00:33:05	00:26:02	02:57:12	01:03:40	01:31:05	05:37:46	28:44:09	33:40:01
**2018-01-13_00000062_1**	00:04:22	00:24:20	00:00:02	00:48:57	01:28:22	05:20:04	28:44:26	37:35:20
**2018-01-13_00000057_1**	00:18:09	00:30:53	00:00:36	00:53:32	01:28:10	05:08:38	29:39:14	32:51:53
**2018-01-13_00000055_1**	00:03:56	00:32:41	00:00:23	00:58:20	01:48:56	05:22:26	35:08:02	32:17:41
**2018-01-13_00000054_1**	00:13:44	00:29:27	00:00:10	00:44:06	01:20:45	04:38:12	30:47:49	30:32:27
**2018-01-13_00000050_1**	00:05:56	00:37:40	00:00:21	00:48:17	01:47:57	05:01:23	36:54:00	38:30:39
**2018-01-13_00000047_1**	00:02:31	00:27:27	00:00:08	00:24:04	01:12:32	03:56:10	32:08:36	41:52:27
**2018-01-13_00000045_2**	00:06:22	00:31:05	02:57:12	00:40:41	01:58:22	02:59:47	33:23:28	44:57:04
**2018-01-13_00000045_1**	00:02:56	00:28:55	00:00:36	00:18:32	00:29:56	01:11:38	20:46:02	17:03:54
**2018-01-13_00000043_1**	00:07:43	00:36:28	00:00:10	00:29:06	00:44:44	00:56:13	30:11:49	21:48:28
**2018-01-13_00000040_1**	01:23:24	00:50:20	00:00:57	01:49:52	00:39:24	04:00:59	50:07:29	44:03:20
**2018-01-13_00000004_1**	00:08:03	00:39:48	00:00:09	00:26:05	00:45:03	01:33:20	34:16:13	24:51:46
**2018-01-06_00000180_1**	00:14:59	00:51:43	00:00:25	01:50:21	01:17:00	24:35:27	40:25:05	74:19:43
**2018-01-06_00000178_1**	00:26:36	00:52:33	00:00:13	01:52:09	01:10:37	20:33:16	39:25:42	72:31:32
**2018-01-06_00000118_1**	00:05:10	00:53:53	02:57:39	01:03:30	00:53:11	13:29:35	28:12:16	63:13:53
**2018-01-06_00000114_1**	00:02:59	00:51:44	02:57:39	00:54:20	01:01:59	13:35:27	29:24:04	63:14:43
**2018-01-06_00000107_1**	00:04:48	00:56:33	00:00:11	00:55:07	00:48:49	11:31:13	28:09:52	60:13:33
**2018-01-06_00000105_1**	00:16:33	01:06:22	00:00:00	01:09:55	01:10:34	10:45:03	34:05:37	67:45:22
**2017-12-23_00000093_1**	00:05:03	00:53:48	02:58:28	01:15:26	01:03:05	10:09:33	40:42:08	37:52:48
**2017-12-23_00000090_1**	00:05:48	00:52:33	00:00:15	00:55:11	00:58:49	08:37:17	40:40:53	36:56:32
**2017-12-23_00000089_1**	00:01:34	00:50:18	00:00:00	00:45:57	00:50:35	07:59:02	22:07:38	36:47:17
**2017-12-23_00000086_1**	00:13:18	01:06:02	02:58:28	01:14:41	01:46:20	08:59:47	50:26:24	37:00:02
**2017-12-23_00000084_1**	00:07:53	01:05:50	00:00:31	01:13:27	00:45:54	09:08:33	50:37:58	40:50:50
**2017-12-23_00000066_1**	00:03:39	00:55:24	00:00:05	00:57:01	00:59:41	08:22:07	43:21:45	28:22:24
**2017-12-23_00000047_1**	00:03:58	00:55:56	00:00:40	00:44:36	00:55:00	06:50:42	41:43:03	38:00:55
**2017-12-23_00000046_1**	00:13:46	01:03:31	00:00:11	00:53:06	00:58:47	07:14:13	45:52:50	36:06:30
**2017-12-23_00000041_1**	00:38:18	01:08:16	02:58:28	00:40:54	00:44:19	05:55:00	50:18:23	24:21:16
**2017-12-23_00000040_1**	00:04:05	00:57:49	02:58:28	00:25:27	00:27:06	04:58:33	37:16:11	25:57:49
**2017-12-23_00000039_1**	00:23:53	01:05:37	00:00:19	00:45:15	01:00:55	06:11:21	50:30:00	36:50:36

**2017-12-23_00000038_1**	00:07:30	01:04:26	00:00:08	00:32:04	00:35:33	05:46:10	46:12:36	29:45:26
**2017-12-23_00000027_1**	00:02:19	01:02:04	02:58:28	00:22:40	00:21:20	06:13:48	38:49:25	23:18:03
**2017-12-23_00000021_2**	00:02:57	01:06:42	00:00:25	00:19:21	00:17:58	03:39:28	41:51:01	17:53:41
**2017-12-23_00000015_1**	00:01:45	01:01:28	00:00:09	00:02:05	00:04:46	02:03:12	16:33:50	27:02:27
**2017-12-23_00000001_1**	00:05:49	00:04:33	02:58:28	00:26:53	00:40:51	02:53:10	43:10:03	26:04:32
**2017-12-16_00000160_1**	01:25:37	00:43:21	00:00:01	01:34:57	01:15:37	06:15:03	32:02:40	49:20:21
**2017-12-16_00000124_1**	00:15:09	00:42:05	02:58:48	01:43:44	01:16:09	06:38:50	29:49:13	37:29:05
**2017-12-16_00000109_1**	00:06:51	00:49:37	00:00:21	01:33:17	01:01:52	10:26:22	47:18:56	38:36:36
**2017-12-16_00000096_1**	01:35:23	00:45:22	00:00:04	01:38:00	01:07:24	05:31:07	30:44:29	38:39:21
**2017-12-16_00000083_1**	00:12:40	00:47:38	00:00:21	01:16:17	01:16:41	05:17:24	49:58:46	31:55:37
**2017-12-16_00000061_1**	00:10:49	00:43:48	00:01:30	01:51:26	00:45:50	04:42:32	56:37:55	55:05:48
**2017-12-16_00000058_1**	00:14:31	00:45:25	00:00:03	00:58:59	01:15:31	06:42:06	38:16:34	37:13:24
**2017-12-16_00000045_2**	00:53:05	00:46:01	02:58:48	00:49:38	00:41:05	03:24:45	29:58:10	26:57:01
**2017-12-16_00000044_2**	00:54:52	00:46:36	00:00:18	00:40:14	00:39:52	03:25:21	29:49:55	21:36:35
**2017-12-16_00000018_1**	00:16:02	00:55:45	00:00:26	01:01:22	00:23:03	02:55:29	52:08:05	26:21:45
**2017-12-16_00000009_1**	01:02:35	00:54:34	00:00:15	00:52:10	01:30:35	01:25:17	36:50:40	20:47:33
**2017-12-16_00000002_1**	00:01:56	00:44:53	02:58:48	00:14:32	00:19:57	00:24:38	25:46:00	07:42:52
**2017-12-16_00000000_1**	00:02:47	00:46:30	02:58:48	00:16:51	00:45:48	02:51:02	34:24:04	27:24:29
**2017-12-09_00000061_1**	00:06:53	05:45:37	00:00:18	01:22:14	01:20:54	16:54:21	27:31:57	65:55:37
**2017-12-09_00000060_1**	00:07:25	05:46:23	00:00:06	01:16:01	01:17:26	19:14:08	29:11:29	65:58:22
**2017-12-09_00000058_1**	00:26:13	05:47:57	02:59:09	01:09:32	01:12:13	15:40:39	36:16:17	68:24:57
**2017-12-09_00000057_1**	00:04:02	05:51:45	00:00:25	01:31:21	01:54:02	14:33:28	33:06:08	38:55:44
**2017-12-09_00000051_1**	00:09:54	05:48:38	00:00:16	01:09:11	01:25:54	13:02:20	31:28:59	36:15:37
**2017-12-09_00000050_1**	00:09:43	05:50:27	00:00:07	01:02:03	01:05:43	12:14:09	29:41:49	35:13:27
**2017-12-09_00000048_1**	00:14:32	05:58:17	02:59:09	01:17:54	01:32:32	14:37:02	33:30:37	33:22:17
**2017-12-09_00000046_1**	00:11:23	05:50:06	02:59:09	00:39:41	01:14:24	10:31:48	32:32:29	21:40:06
**2017-12-09_00000040_1**	00:05:16	05:51:00	02:59:09	00:44:33	00:46:16	14:42:43	46:59:21	76:26:59
**2017-12-09_00000037_1**	00:01:49	05:45:47	02:59:09	00:29:25	00:47:49	08:15:32	23:50:53	11:54:47
**2017-12-09_00000021_1**	00:02:09	05:45:53	02:59:09	00:14:26	00:23:10	06:26:34	18:46:15	36:37:52
**2017-12-09_00000018_1**	00:59:47	06:09:43	00:00:23	00:52:19	01:19:47	06:36:26	37:24:52	19:41:42
**2017-12-09_00000015_1**	00:11:31	06:15:23	00:01:00	02:16:56	00:29:32	05:47:02	49:15:38	33:13:23
**2017-12-09_00000010_2**	00:38:10	05:55:08	02:59:09	00:14:42	00:16:10	02:26:51	24:39:16	10:11:07
**2017-12-09_00000008_2**	00:01:58	05:51:43	00:00:24	00:07:20	00:09:58	02:04:27	23:11:03	05:34:43
**2017-12-09_00000005_1**	00:02:22	05:54:06	02:59:09	00:04:41	00:04:23	01:32:50	17:31:27	17:03:06
**2017-12-09_00000004_1**	00:02:25	05:55:10	02:59:09	00:04:31	00:05:26	01:07:46	21:21:35	21:59:09

**2017-12-02_00000122_1**	00:02:36	00:38:19	00:00:02	01:39:57	01:05:37	14:29:05	24:00:42	20:39:18
**2017-12-02_00000116_1**	00:15:10	00:46:07	02:59:25	01:50:46	01:59:11	14:41:52	30:46:16	25:15:06
**2017-12-02_00000114_1**	02:25:01	00:59:44	00:00:24	02:10:20	01:03:02	15:26:27	45:22:05	76:27:44
**2017-12-02_00000109_1**	00:09:32	00:40:31	02:59:25	01:05:09	00:48:33	12:59:15	23:01:38	31:02:30
**2017-12-02_00000106_1**	00:51:22	00:46:06	02:59:25	01:15:42	01:00:23	13:27:49	27:32:28	25:54:05
**2017-12-02_00000102_1**	00:13:11	00:40:56	02:59:25	01:07:32	01:05:12	12:21:39	27:55:15	35:16:55
**2017-12-02_00000078_1**	00:12:44	00:39:40	00:00:23	00:57:19	00:53:45	12:17:26	27:01:50	26:21:40
**2017-12-02_00000076_1**	00:09:31	00:40:15	02:59:25	00:53:54	00:51:32	10:18:00	26:54:35	27:20:15
**2017-12-02_00000071_1**	00:13:19	00:48:02	02:59:25	01:03:39	01:17:20	10:30:46	33:01:23	34:23:02
**2017-12-02_00000070_1**	00:43:55	00:50:52	00:00:31	01:05:27	01:25:56	10:54:33	34:57:01	28:36:52
**2017-12-02_00000069_1**	00:05:46	00:43:29	00:00:10	01:16:05	00:36:47	07:59:12	35:57:50	31:50:29
**2017-12-02_00000058_1**	00:06:09	00:42:52	00:00:34	00:35:30	00:47:10	05:40:37	28:32:13	14:19:51
**2017-12-02_00000048_1**	00:25:46	00:56:29	00:00:10	00:39:06	00:49:47	04:49:12	30:28:51	24:43:29
**2017-12-02_00000042_2**	00:05:20	00:49:18	02:59:25	00:16:55	00:30:21	03:52:02	27:35:24	23:27:17
**2017-12-02_00000042_1**	00:05:07	00:49:51	02:59:25	00:21:28	00:32:08	02:33:35	29:49:11	25:14:51
**2017-12-02_00000030_1**	00:06:31	00:49:14	02:59:25	00:22:51	00:29:32	01:43:58	29:59:36	09:49:13
**2017-12-02_00000023_1**	00:04:37	00:50:20	02:59:25	00:23:40	00:33:38	01:58:52	27:12:48	19:55:19
**2017-11-25_00000069_1**	00:04:23	00:45:07	02:59:50	15:34:43	01:49:24	16:20:50	24:32:29	37:11:07
**2017-11-25_00000066_1**	00:10:59	00:50:56	00:00:36	15:34:33	01:43:00	28:11:39	40:34:05	46:41:55
**2017-11-25_00000061_1**	00:06:44	00:49:29	00:00:12	15:15:09	01:32:44	13:48:14	33:01:47	37:06:28
**2017-11-25_00000060_1**	00:17:29	00:52:14	02:59:50	15:09:51	02:28:30	13:23:59	31:56:33	36:51:14
**2017-11-25_00000056_1**	00:07:03	00:51:01	02:59:50	15:03:37	01:43:04	11:31:44	29:39:08	36:38:00
**2017-11-25_00000055_1**	00:07:51	00:49:34	00:00:15	14:58:12	01:21:51	11:54:18	32:35:54	39:43:33
**2017-11-25_00000050_1**	00:23:15	00:56:59	00:00:38	14:49:34	01:12:15	10:21:42	35:01:20	37:45:58
**2017-11-25_00000043_1**	00:07:02	00:57:46	00:00:28	14:42:24	01:23:02	09:11:31	26:33:05	20:04:45
**2017-11-25_00000041_1**	00:06:48	00:57:33	00:00:13	14:42:09	01:01:48	08:40:16	41:27:51	30:19:32
**2017-11-25_00000035_1**	00:15:56	01:05:55	00:00:36	14:34:32	00:40:57	07:52:39	52:37:00	37:03:55
**2017-11-25_00000030_1**	00:07:43	00:56:27	00:00:08	14:28:04	00:41:44	07:32:11	25:36:47	19:56:27
**2017-11-25_00000022_1**	00:13:32	01:02:15	02:59:50	14:27:51	00:24:33	04:04:59	39:02:36	24:31:15
**2017-11-25_00000021_1**	00:04:27	00:56:05	02:59:50	11:52:40	00:29:28	01:59:48	24:36:33	09:23:04
**2017-11-25_00000020_1**	00:03:17	00:59:02	02:59:50	11:31:38	00:24:18	02:11:46	26:23:22	06:44:01
**2017-11-25_00000018_1**	00:06:03	01:06:48	00:00:31	11:10:27	00:47:04	01:52:33	32:06:07	11:23:48
**2017-11-25_00000017_1**	00:02:52	01:00:35	00:00:15	11:11:11	00:05:53	01:10:19	21:02:57	15:09:35
**2017-11-25_00000007_1**	00:07:53	01:01:38	00:00:06	11:10:01	00:27:54	04:14:15	29:08:01	16:21:37
**2017-11-18_00000073_1**	00:07:05	00:12:50	03:00:06	00:44:28	01:16:06	09:05:36	20:19:11	48:40:50

**2017-11-18_00000069_1**	00:06:39	00:13:37	03:00:06	00:47:14	01:10:39	06:53:21	20:50:43	48:23:36
**2017-11-18_00000068_1**	00:09:13	00:25:10	03:00:06	01:05:47	01:34:13	07:15:54	32:41:17	42:15:09
**2017-11-18_00000014_1**	01:01:39	00:24:36	00:00:19	00:36:15	01:39:39	04:59:22	33:20:44	39:53:36
**2017-11-18_00000009_1**	00:15:29	00:26:12	00:00:52	01:02:48	00:36:29	06:34:54	33:57:32	34:42:11
**2017-11-11_00000093_1**	00:18:33	00:22:25	00:00:05	00:43:01	01:33:33	04:45:09	20:29:39	62:48:24
**2017-11-11_00000092_1**	00:18:11	00:22:09	03:00:25	00:34:42	01:33:12	06:25:49	19:37:22	62:49:08
**2017-11-11_00000086_1**	00:30:09	00:31:52	03:00:25	00:43:26	01:36:10	04:05:34	22:57:14	62:54:51
**2017-11-11_00000077_1**	00:27:47	00:27:43	00:00:22	01:15:18	01:27:49	07:21:24	32:03:55	62:57:43
**2017-11-11_00000070_1**	00:13:40	00:25:22	00:00:03	00:36:59	01:21:41	03:33:06	21:50:44	62:51:22
**2017-11-11_00000057_1**	00:14:48	00:30:45	00:00:25	01:26:21	01:16:49	04:59:28	35:17:54	63:02:45
**2017-11-11_00000054_1**	00:31:22	00:30:20	00:00:02	00:37:58	01:11:22	03:40:04	23:21:28	62:55:19
**2017-11-11_00000052_1**	00:26:01	00:39:44	00:00:26	00:55:22	01:24:01	03:05:28	29:04:07	63:04:44
**2017-11-11_00000036_1**	00:25:28	00:37:11	03:00:25	00:36:49	00:53:28	02:22:54	22:19:39	63:01:11
**2017-11-11_00000027_2**	00:16:21	00:35:05	03:00:25	00:30:42	00:41:22	02:00:50	20:30:27	63:02:05
**2017-11-11_00000026_2**	00:16:54	00:35:51	03:00:25	00:31:31	00:34:55	01:56:37	20:32:03	62:57:51
**2017-11-11_00000025_2**	00:17:33	00:36:28	00:00:10	00:27:07	00:29:34	01:32:14	20:31:40	63:03:28
**2017-11-11_00000024_2**	00:18:21	00:37:04	03:00:25	00:24:44	00:23:21	02:06:50	21:07:32	63:04:04
**2017-11-11_00000020_1**	00:38:18	00:49:00	00:00:40	00:30:36	00:38:19	02:01:44	35:52:33	63:07:00
**2017-11-11_00000016_2**	00:02:25	00:34:04	03:00:25	00:04:37	00:14:25	00:25:45	18:10:42	63:06:03
**2017-11-11_00000010_1**	00:31:27	00:46:09	03:00:25	00:28:46	00:36:28	01:03:53	28:40:42	63:10:09
**2017-11-11_00000006_1**	00:05:44	00:40:21	03:00:25	00:24:46	00:26:45	01:42:59	26:41:26	63:09:20
**2017-11-04_00000068_1**	00:10:27	00:08:08	03:00:44	01:07:47	01:20:27	04:48:55	38:56:31	35:41:08
**2017-11-04_00000055_2**	00:26:01	00:09:58	03:00:44	01:03:35	01:07:02	04:12:42	32:04:06	36:01:57
**2017-11-04_00000048_1**	00:07:33	00:08:17	03:00:44	00:40:49	01:03:33	03:28:58	28:14:39	44:41:17
**2017-11-04_00000042_1**	00:09:01	00:12:46	03:00:44	00:42:24	01:08:02	03:37:31	31:58:08	26:22:45
**2017-11-04_00000037_1**	00:10:41	00:17:36	00:00:17	00:40:12	01:15:41	04:06:19	64:59:49	37:41:36
**2017-11-04_00000032_1**	00:17:09	00:23:03	03:00:44	00:52:42	01:39:10	03:10:48	61:59:18	24:53:02
**2017-11-04_00000023_2**	00:21:03	00:21:46	00:00:26	00:32:22	01:05:04	02:44:30	61:34:09	20:07:45
**2017-11-04_00000020_2**	00:22:33	00:23:18	00:00:00	00:28:56	00:54:34	02:28:03	63:57:48	16:53:17
**2017-11-04_00000013_1**	00:12:18	00:18:57	00:00:37	00:34:34	01:12:19	01:48:41	60:52:36	22:10:56
**2017-11-04_00000009_1**	00:03:34	00:17:05	03:00:44	00:23:40	00:48:35	00:59:49	59:50:56	20:27:04
**2017-10-28_00000134_1**	00:12:24	00:44:07	03:01:07	01:16:46	20:29:24	05:47:52	23:08:30	32:41:07
**2017-10-28_00000126_1**	00:17:48	00:53:45	00:00:29	01:40:25	20:56:48	06:30:31	41:52:53	38:20:45
**2017-10-28_00000119_1**	00:22:34	00:52:17	00:00:01	01:17:57	20:38:34	08:44:03	31:55:37	45:04:16
**2017-10-28_00000109_1**	00:28:08	01:00:03	00:00:45	01:38:41	21:08:08	05:28:48	42:47:12	38:30:02

**2017-10-28_00000099_2**	00:05:52	00:45:37	03:01:07	01:00:16	20:17:53	05:39:22	31:45:57	38:20:37
**2017-10-28_00000075_1**	00:08:38	00:49:21	00:00:05	01:00:01	20:05:38	04:12:07	27:22:42	33:45:21
**2017-10-28_00000074_1**	00:14:09	00:51:07	03:01:07	01:22:47	20:28:10	05:30:53	37:29:15	37:37:06
**2017-10-28_00000068_1**	00:03:57	00:48:39	03:01:07	01:00:19	19:39:57	06:48:25	27:21:01	33:06:39
**2017-10-28_00000067_1**	00:06:28	00:48:26	00:00:09	00:58:05	19:31:28	03:44:12	27:22:35	37:06:25
**2017-10-28_00000062_1**	00:11:02	00:52:47	03:01:07	00:46:26	18:44:02	02:13:32	20:49:07	11:41:46
**2017-10-28_00000061_1**	00:11:34	00:56:32	00:00:15	00:46:11	18:44:34	02:39:17	29:31:37	20:05:31
**2017-10-28_00000052_1**	00:05:22	00:52:03	03:01:07	00:37:41	16:47:22	01:51:49	29:12:26	11:36:03
**2017-10-28_00000044_1**	00:17:01	01:07:54	00:00:37	01:01:33	15:52:01	03:55:40	37:22:05	45:39:54
**2017-10-28_00000041_1**	00:13:33	00:56:30	00:00:13	00:46:09	11:43:33	01:20:16	32:41:38	20:58:29
**2017-10-28_00000029_1**	00:08:20	00:57:04	03:01:07	00:15:42	09:27:21	00:24:50	27:27:27	08:42:04
**2017-10-28_00000027_2**	00:08:16	00:58:54	00:00:36	01:02:31	04:33:17	02:06:39	41:58:24	36:19:54
**2017-10-28_00000021_1**	00:23:08	01:15:50	00:00:33	01:18:29	02:16:08	04:48:36	47:20:12	36:56:50
**2017-10-21_00000060_2**	00:07:50	00:41:34	03:01:28	01:08:11	01:21:51	07:19:20	27:32:54	50:22:34
**2017-10-21_00000059_1**	00:31:28	00:48:25	00:00:02	01:10:58	01:16:28	06:58:04	27:20:32	49:17:25
**2017-10-21_00000051_1**	00:11:15	00:47:58	03:01:28	01:18:36	01:23:16	06:17:44	30:05:18	49:17:57
**2017-10-21_00000036_1**	00:16:57	00:49:43	00:00:26	01:07:22	01:05:58	05:31:28	26:57:03	49:15:42
**2017-10-21_00000034_1**	00:19:31	00:50:29	00:00:11	01:03:07	00:59:31	06:21:14	25:42:35	49:17:28
**2017-10-21_00000032_1**	00:06:46	00:45:45	03:01:28	00:51:23	00:51:47	04:33:31	16:31:49	32:44:44
**2017-10-21_00000031_1**	00:07:29	00:45:13	03:01:28	00:48:53	00:52:29	04:59:59	17:37:33	36:33:13
**2017-10-21_00000029_1**	00:04:16	00:45:59	03:01:28	00:45:37	00:48:17	04:25:44	12:34:20	32:13:59
**2017-10-21_00000026_1**	00:03:01	00:46:45	03:01:28	00:35:24	00:45:01	04:24:30	12:17:05	37:10:44
**2017-10-21_00000023_1**	00:04:52	00:50:31	00:00:14	00:42:09	00:51:53	03:56:17	24:02:55	30:16:31
**2017-10-21_00000022_1**	00:13:38	00:55:21	00:00:03	00:57:59	01:14:38	04:49:06	29:04:42	33:39:20
**2017-10-21_00000020_1**	00:08:25	00:52:09	03:01:28	00:26:46	00:35:25	02:20:54	19:25:29	19:37:08
**2017-10-21_00000019_1**	00:14:57	01:05:55	00:00:39	01:56:33	00:41:57	05:34:41	46:02:00	37:23:55
**2017-10-21_00000015_1**	00:20:41	01:07:26	00:00:09	01:04:04	01:32:41	04:08:11	33:36:44	34:45:25
**2017-10-21_00000012_1**	00:08:18	00:55:09	03:01:28	00:27:48	00:23:18	17:20:55	34:16:22	40:33:09
**2017-10-21_00000006_1**	00:34:06	01:03:49	03:01:28	00:21:26	00:39:07	01:48:35	31:07:12	26:16:49
**2017-10-21_00000005_1**	00:09:59	00:59:44	00:00:22	00:35:16	00:55:59	02:01:25	30:50:02	36:56:43
**2017-10-21_00000002_1**	00:06:05	00:56:49	00:00:10	00:18:06	00:18:05	02:04:19	24:28:13	16:57:46

Supplementary Table 1 Raw data for "Response Time" column of Table 1. Target ID refers to the CAMEO target.

Raw data – lDDT Easy

Target	SWISS-MODEL	HHpredB	NaiveBLAST	PRIMO	SPARKS-X	RaptorX	IntFOLD4-TS	Robetta
2018-01-13_00000066_2	82,45	79,97	71,63	83,57	75,85	81,44	81,7	82,47
2018-01-13_00000057_1	88,01	84,4	86,2	87,11	80,52	86,69	83,35	87,88
2018-01-13_00000055_1	80,56	81,37	81,04	83,76	79,62	82,33	83,93	84,61
2018-01-13_00000050_1	85,74	73,5	83,95	84,11	78,29	80,85	79,85	78,43
2018-01-13_00000043_1	91,47	77,1	87,71	89,21	71,78	84,51	88,85	88,01
2018-01-06_00000105_1	84,38	81,47	84,02	84,75	81,98	82,89	81,45	86,47
2017-12-23_00000086_1	81,4	78,34	79,34	79,98	80,24	80,81	81,76	80,07
2017-12-23_00000084_1	81,54	81,41	78,35	80,26	77,79	78,28	79,45	78,62
2017-12-23_00000066_1	85,05	81,07	83,28	83,76	80,72	83,04	83,81	84,5
2017-12-23_00000046_1	85,95	75,18	81,82	77,92	77,15	86,17	86,23	81,95
2017-12-16_00000045_2	83,19	82,35	82,41	81,58	76,74	84,35	78,37	84,34
2017-12-16_00000018_1	82,38	74,36	79,04	79,49	77,27	79,14	78,83	82,09
2017-12-09_00000057_1	93,08	90,1	86,97	91,59	89,98	88,93	91,01	88,66
2017-12-09_00000048_1	86,27	86,05	82,63	84,89	81,34	83,2	82,25	85,74
2017-12-09_00000037_1	87,23	80,22	84,35	85,77	83,45	80,16	84,14	83,71
2017-12-09_00000018_1	86,33	83,86	83,23	85,52	71,27	82,18	82,7	79,87
2017-12-09_00000015_1	88,96	86,11	89,89	89,81	89,67	86,04	89,24	86,61
2017-11-25_00000022_1	84,37	80,92	80,73	81,03	81,5	81,91	82,79	83,77
2017-11-25_00000020_1	84,28	84,81	83,17	83,23	83,5	84,31	82,64	89,25
2017-11-25_00000018_1	89,06	83,46	86,35	86,8	74,51	85,89	85,02	86,79
2017-11-18_00000009_1	89,34	85,35	80,87	81,49	86,5	79,85	80,68	83,79
2017-11-11_00000070_1	91,89	84,46	86,25	88,19	81,44	82,75	86,82	87,81
2017-11-11_00000027_2	83,13	82,07	81,11	81,57	71,79	81,24	77,96	85,35
2017-11-11_00000026_2	82,18	83,43	80,35	80,8	69,29	81,98	79,57	84,84
2017-11-11_00000025_2	83,02	75,36	81,46	81,65	74,77	77,96	78,6	86,31
2017-11-11_00000006_1	87,66	83,48	85,78	86,9	84,7	84,86	83,08	86,06
2017-11-04_00000068_1	81,91	81,07	78,04	78,62	75,26	81,02	80,92	85,52
2017-11-04_00000032_1	87,53	75,34	88,99	91,01	80,83	86,88	88,87	87,7
2017-10-28_00000052_1	77,2	76,85	78,24	79,6	75,33	77,7	78,12	80,53
2017-10-28_00000044_1	91,96	86,05	87,58	88,34	80,69	87,12	86,7	88,16
2017-10-28_00000041_1	95,31	88,74	91,06	92,79	89,81	86,67	90,46	84,73
2017-10-21_00000036_1	80,09	79,95	72,42	79,74	73,61	84,77	82,33	84,52

**2017-10-21_00000034_1**	80,22	79,34	77,68	80,73	76,29	85,62	84,7	85,38
**2017-10-21_00000022_1**	95,15	90,47	90,63	92,65	92,34	90,72	91,93	92,04
**2017-10-21_00000020_1**	88,23	86,61	80,88	85,13	83,22	83,9	85,33	87,93
**2017-10-21_00000015_1**	89,91	85,65	87,54	88,61	87,43	82,98	87,97	87,6
**2017-10-21_00000002_1**	86	87,53	80,84	84,87	85,92	84,68	87,89	89,3

Supplementary Table 2. Raw data for "lDDT Easy" column of Table 1. Target ID refers to the CAMEO target. Please note that the data for the "lDDT Total" column of Table 1 is the union of "lDDT Easy", "lDDT Medium" and "lDDT Hard".

Raw data – lDDT Medium

Target	SWISS-MODEL	HHpredB	NaiveB LAST	PRIMO	SPARKS-X	RaptorX	IntFOLD 4-TS	Robetta
2018-01-13_00000090_1	63,64	60,41	34,82	35,09	69,06	70,85	69,03	71,36
2018-01-13_00000062_1	64,87	59,13	53,98	50,65	52,2	61,61	59,16	63,62
2018-01-13_00000054_1	81,04	80,93	78,14	80,85	77,37	79,3	77,76	81,51
2018-01-13_00000045_2	69,7	67,44	68,1	67,42	59,78	65,51	59,31	66,83
2018-01-13_00000045_1	72,47	75,04	73,75	72,6	71,16	72,54	74,77	73,92
2018-01-13_00000040_1	79,69	75,2	68,68	69,09	68,51	68,25	70,94	75,15
2018-01-13_00000004_1	65,62	67,22	42,89	60,64	65,91	72,99	69,49	73,52
2018-01-06_00000107_1	90,07	63,21	83,8	56,54	62,85	86,45	83,72	73,28
2017-12-23_00000093_1	71,88	69,79	60,45	63,96	70,77	69,39	71,77	75,63
2017-12-23_00000090_1	70,31	70,79	68,15	69,78	68,2	70,31	68,43	71,38
2017-12-23_00000089_1	75,72	74,42	70,56	73,6	71,87	73,62	74,81	74,86
2017-12-23_00000041_1	72,84	80,86	73,54	74,06	66,16	76,96	77,27	85,07
2017-12-23_00000040_1	54,78	77,99	72,14	73,24	52,63	55,75	61,23	80,87
2017-12-23_00000039_1	58,44	65,84	55,77	57,28	56,14	65,75	66,48	70,32
2017-12-23_00000038_1	72,16	73,16	67,75	67,52	69,77	70,6	69,28	75,1
2017-12-23_00000021_2	69,17	68,18	63,07	65,5	65,55	68,71	68,41	73,07
2017-12-16_00000160_1	64,86	64,72	49,26	50,15	65,02	79,7	66,69	67,16
2017-12-16_00000124_1	79,9	71,27	77,32	79,02	73,29	79,02	80,41	82,75
2017-12-16_00000109_1	67,5	69,05	62,43	64,33	68,67	68,67	69,01	65,85
2017-12-16_00000096_1	66,06	67,17	63,91	67,47	68,69	74,37	67,53	74,98
2017-12-16_00000083_1	73,43	72,89	72,28	72,91	55,69	71,7	71,7	73,39
2017-12-16_00000061_1	63,42	59,25	56,71	58,52	59,68	60,92	62,22	44,62
2017-12-16_00000044_2	84,77	82,72	83,82	82,87	77,73	85,78	77,49	87,09
2017-12-16_00000009_1	74,09	74,26	72,64	75,56	71,06	74,41	72,32	79,44
2017-12-16_00000002_1	76,09	76,57	75,86	76,51	73,88	74,94	73,25	77,82
2017-12-09_00000061_1	52,52	57,9	50,49	52,95	51,54	59,26	56,26	61,69
2017-12-09_00000051_1	85	83,2	81,47	84,36	73,12	83,35	83,93	86,68
2017-12-09_00000010_2	79,71	77,15	80,82	82,81	66,64	84,32	74,92	83,04
2017-12-09_00000008_2	68	74,22	47,95	66,84	62,54	69,07	68,23	76,23
2017-12-09_00000005_1	65,66	78,15	76,91	81,88	67,56	72,56	77,58	79,71
2017-12-09_00000004_1	52,96	70,61	62,17	64,03	58,01	69,15	68,94	65,3
2017-12-02_00000122_1	86,43	82,75	75,82	80,46	66,26	81,02	85,48	84,83

**2017-12-02_00000116_1**	75,66	85,32	90,47	93,33	88,35	87,32	91,02	80,74
**2017-12-02_00000109_1**	57,68	60,7	53,05	44	68,76	60,61	70,03	73,54
**2017-12-02_00000106_1**	84,74	81,79	82,56	83,83	79,18	85,22	82,47	78,87
**2017-12-02_00000076_1**	56,93	51,26	40,39	48,72	55,93	59,18	60,04	58,93
**2017-12-02_00000070_1**	79,72	76,9	75,85	71,95	69,45	73,39	77,89	78,11
**2017-12-02_00000069_1**	67,89	66,12	65,52	66,18	65,96	66,86	66,06	74,06
**2017-12-02_00000058_1**	69,28	62,96	80,62	64,2	67,16	82,23	68,29	85,14
**2017-12-02_00000048_1**	65,16	63,56	55,78	63,11	62,26	66,3	62,17	70,88
**2017-12-02_00000030_1**	67,67	61,19	81,9	65,73	66,11	84,82	66,15	87,34
**2017-12-02_00000023_1**	61,42	58,68	39,36	49,77	60,35	65,48	62,06	70,45
**2017-11-25_00000061_1**	62,25	54,16	56,87	60,07	47,95	63,04	59,04	58,24
**2017-11-25_00000060_1**	71,44	68,32	60,65	64,47	65,83	71,29	70,62	71,18
**2017-11-25_00000056_1**	54,28	56,16	51,01	52,19	52,9	57,98	58,11	62,41
**2017-11-25_00000055_1**	95,76	61,37	92,44	57,74	59,16	90,06	91,57	84,2
**2017-11-25_00000050_1**	69,76	67,4	59,98	61,6	66,86	64,87	68,32	72,59
**2017-11-25_00000043_1**	73,35	76,56	71,33	71,03	71,63	75,69	73,99	81,04
**2017-11-25_00000041_1**	70,84	69,65	69,98	69,75	69,28	64,06	70,18	70,01
**2017-11-25_00000030_1**	65,42	57,79	35,61	59,1	63,37	64,72	62,32	61,82
**2017-11-25_00000017_1**	49,3	61,86	40,56	63,29	57,24	66,05	62,55	62,51
**2017-11-25_00000007_1**	69,96	62,7	45,28	67,8	67,93	68,21	69,29	71,32
**2017-11-18_00000073_1**	72,16	75,96	22,04	28,01	67,02	70,16	77,98	75,14
**2017-11-18_00000069_1**	81,16	79,83	70,16	77,34	73,33	78,64	78,9	78,61
**2017-11-18_00000068_1**	74,8	71,81	72,21	71,79	73,39	73,73	76,99	78,92
**2017-11-18_00000014_1**	37,69	70,78	44,45	56,98	65,3	60,84	64,62	64,46
**2017-11-11_00000092_1**	63,64	78,4	70,78	79,59	71,17	78,01	75,99	80,1
**2017-11-11_00000086_1**	67,93	65,68	54,31	55,13	64,69	75,34	75,19	71,77
**2017-11-11_00000057_1**	55,45	54,46	43,16	47,59	51,45	55,1	51,71	55,8
**2017-11-11_00000052_1**	61,63	63,35	53,13	57,4	66,6	66,6	69,31	68
**2017-11-11_00000036_1**	83,01	78,72	80,81	82,07	77,81	79,72	79,55	81,91
**2017-11-11_00000024_2**	79,21	73,4	66,46	66,3	67,75	78,53	74,07	78,33
**2017-11-11_00000016_2**	50,9	68,3	50,79	59,94	71,71	77,13	73,62	67,35
**2017-11-11_00000010_1**	56,78	64,24	55,79	59,3	58,97	67,4	65,55	71,32
**2017-11-04_00000055_2**	76,77	76,77	78,15	78,01	72,25	78,57	78,15	78,77
**2017-11-04_00000048_1**	69,24	70,63	66,13	70,51	69,39	71,35	71,43	69,99

**2017-11-04_00000042_1**	75,97	76,54	74,7	75	75,35	75,72	74,74	82,82
**2017-11-04_00000037_1**	72,86	72,37	70,83	62,86	72,25	74,78	73,65	74,26
**2017-11-04_00000023_2**	85,65	82,87	82,53	47	70,16	85,48	77,9	89,11
**2017-11-04_00000020_2**	86,3	80,81	81,67	45,83	68,65	84,16	80,94	86,48
**2017-11-04_00000013_1**	77,65	74,26	74,52	76,52	69,94	77,44	75,82	77,47
**2017-11-04_00000009_1**	67,49	42,19	65,78	18,11	21,61	66,89	66,32	65,61
**2017-10-28_00000134_1**	61,18	70,56	60,65	59,57	66,38	70,11	62,24	73,55
**2017-10-28_00000126_1**	84,49	69,52	81,82	84,37	46,57	83,39	83,86	82,44
**2017-10-28_00000109_1**	68,27	51,41	65,88	66,26	64,62	68,8	67,96	71,01
**2017-10-28_00000099_2**	90,35	80,96	86,66	90,04	52,17	66,25	39,89	61,44
**2017-10-28_00000067_1**	59,63	66,11	32,52	69,51	55,69	69,76	63,28	74,32
**2017-10-28_00000062_1**	78,51	82,01	79,29	77,22	74,85	81,04	81,68	82,08
**2017-10-28_00000061_1**	52,45	73,48	46,84	50,05	72,25	70,22	75,09	78,85
**2017-10-28_00000029_1**	78,54	77,92	76,96	78,46	72,45	79,71	80,57	80,06
**2017-10-28_00000021_1**	73,66	71,78	68,5	72,11	65,37	73,7	74,13	74,13
**2017-10-21_00000059_1**	70,06	67,59	35,65	36,51	69,12	69,57	73,07	75,45
**2017-10-21_00000051_1**	64,07	66,52	63,81	60,89	61,61	62,54	65,4	63,77
**2017-10-21_00000032_1**	66,98	65,27	66,62	66,85	65,66	70,02	66,91	79,41
**2017-10-21_00000031_1**	64,38	76,53	49,19	49,86	77,14	77,46	75,26	80,5
**2017-10-21_00000029_1**	70,36	72,63	68,2	68,88	72,65	70,34	74,76	75,33
**2017-10-21_00000026_1**	52,02	59,05	49,65	55,23	58,52	63,17	65,64	69,9
**2017-10-21_00000023_1**	50,75	51,76	51,88	51,83	51,16	52,69	52,54	52,96
**2017-10-21_00000019_1**	73,95	73,11	65,94	70,43	64,76	72,79	71,14	76,56
**2017-10-21_00000006_1**	76,91	77,54	74,48	77,02	68,81	77,51	73,71	82,19

Supplementary Table 3. Raw data for "lDDT Medium" column of Table 1. Target ID refers to the CAMEO target. Please note that the data for the "lDDT Total" column of Table 1 is the union of "lDDT Easy", "lDDT Medium" and "lDDT Hard".

Raw data – IDDT Hard

Target	SWISS-MODEL	HHpredB	NaiveBLAST	PRIMO	SPARKS-X	RaptorX	IntFOLD4-TS	Robetta
2018-01-13_00000073_1	62,4	47,6	20,88	20,89	46,75	49,74	46,67	56,39
2018-01-13_00000068_1	60,42	50,51	22	22,51	45,66	51,62	45,45	59,64
2018-01-13_00000047_1	5,82	18,22	2,03	3,05	39,18	41,73	34,68	41,86
2018-01-06_00000180_1	43,01	40,44	12,92	30,81	41,52	43,3	46,97	45,19
2018-01-06_00000178_1	46,78	45,09	13,01	31,85	45,14	45,09	48,35	49,09
2018-01-06_00000118_1	31,88	32,91	6,49	23,17	34,04	35,95	35,43	37,17
2018-01-06_00000114_1	42,36	42,32	19,89	14,51	41,69	49,71	45,25	55,56
2017-12-23_00000047_1	47,66	42,85	25,77	38,69	43,65	49,61	55,61	59,46
2017-12-23_00000027_1	44,43	65,83	25,46	34,65	51,45	65,67	59,78	64,52
2017-12-23_00000015_1	36,6	40,4	27,65	27,88	36,8	46,24	38,51	44,52
2017-12-23_00000001_1	3,6	33,14	73,37	13,31	15,85	76,37	72,28	76,99
2017-12-16_00000058_1	52,52	51,95	12,77	28,14	48,48	56,37	56,47	54,36
2017-12-16_00000000_1	9,83	34,67	4,02	16,05	20,56	34,22	34,71	22,07
2017-12-09_00000060_1	54,85	56,44	49,71	52,09	50,1	56,12	55,85	59,56
2017-12-09_00000058_1	27,43	50,15	24,54	25,74	54,66	57,91	51,93	59,04
2017-12-09_00000050_1	45,33	48,92	31,78	35,11	38,96	55,55	47,02	54,79
2017-12-09_00000046_1	75,7	49,76	73,01	9,22	46,77	54,08	68,99	75,84
2017-12-09_00000040_1	27,93	22,49	27,2	8,12	25,91	43,18	45,6	49,04
2017-12-09_00000021_1	40,01	45	15,64	19,83	50,06	55,07	50,68	43,61
2017-12-02_00000114_1	26,96	35,61	18,98	27,37	35,84	38,86	35,79	49,07
2017-12-02_00000102_1	32,72	49,57	42,85	47,02	49,08	53,87	49,79	58,09
2017-12-02_00000078_1	55,66	54,88	28,61	49,9	52,76	56,91	53,17	60,06
2017-12-02_00000071_1	51,54	54,37	15,69	47,72	56,68	56,7	55,43	62,81
2017-12-02_00000042_2	48,79	59,65	46,92	50,21	63	61,5	63,98	68,38
2017-12-02_00000042_1	58,21	54,57	46,73	57,94	59,19	63,56	61,37	66,47
2017-11-25_00000069_1	51,83	48,96	31,6	47,45	51,61	59,89	56,54	63,15
2017-11-25_00000066_1	43,2	23,72	22,42	4,3	28,86	49,61	48,52	55,23
2017-11-25_00000035_1	41,59	38,49	15,43	26,74	40,04	43,35	38,9	45,07
2017-11-25_00000021_1	23,05	45,39	22,25	22,16	43,87	36	59,59	47,28
2017-11-11_00000093_1	16,81	23,12	8,12	24,38	27,04	31,03	27,72	56,72
2017-11-11_00000077_1	39,93	35,23	14,54	24,21	33,95	28,83	48,31	44,5
2017-11-11_00000054_1	55,98	66,07	22,3	22,81	57,23	64,59	63,5	67,85

**2017-11-11_00000020_1**	65,04	49,84	63,41	21,71	54,95	64,7	64,97	70,38
**2017-10-28_00000119_1**	26,5	31,36	11,55	20,56	34,41	38,43	35,78	40,25
**2017-10-28_00000075_1**	59,87	37,2	58,45	15,08	46,71	62,93	60,01	63,56
**2017-10-28_00000074_1**	36,57	46,4	1,87	15,03	42,16	46,46	51,13	49,86
**2017-10-28_00000068_1**	52,62	56,44	27,53	52,28	51,88	62,01	59,76	61,27
**2017-10-28_00000027_2**	39,45	42,56	33,44	41,32	39,73	43,46	40,19	43,13
**2017-10-21_00000060_2**	6,17	18,79	7,64	20,64	23,62	26,58	26,64	51,97
**2017-10-21_00000012_1**	29,1	34,25	1,25	17,14	32,1	42,33	37,75	36,12
**2017-10-21_00000005_1**	47,51	45,3	26,31	28,84	41,64	45,93	34,47	47,46

Supplementary Table 4. Raw data for "lDDT Hard" column of Table 1. Target ID refers to the CAMEO target. Please note that the data for the "lDDT Total" column of Table 1 is the union of "lDDT Easy", "lDDT Medium" and "lDDT Hard".

Raw data – IDDT BS

Target	SWISS-MODEL	HHpred B	NaiveBLA ST	PRIMO	SPARKS-X	Raptor X	IntFOLD4-TS	Robetta
2018-01-13_00000057_1	67,88	75,94	57,74	65,09	59,29	74,1	74,62	75,57
2018-01-13_00000055_1	100	96,92	93,57	91,49	98,1	97,37	98,19	89,13
2018-01-13_00000054_1	44,84	41,65	41,63	42,65	42,74	41,29	41,37	40,29
2018-01-13_00000043_1	60,7	67,09	62,86	60,61	62,24	65,67	67,63	63,75
2018-01-13_00000040_1	50,86	50,44	45,33	44,28	44,64	43,4	62,31	52,01
2018-01-13_00000004_1	68,79	71,2	61,2	56,51	73,49	71,59	71,19	68,23
2018-01-06_00000180_1	57,36	61,27	34,76	54,33	59,54	55,54	63,17	58,91
2018-01-06_00000178_1	66,47	72,9	41,33	53,71	68,97	71,84	71,68	63,61
2018-01-06_00000118_1	86,36	89,77	0	19,6	81,39	78,84	45,74	69,46
2018-01-06_00000114_1	13,13	30,4	9,75	3,8	27,61	25,12	27,08	32,53
2018-01-06_00000107_1	53,99	48,1	50,47	45,26	43,29	49,81	48,6	47,08
2018-01-06_00000105_1	63,55	58,22	64,22	63,69	53,58	57,36	64,62	59,96
2017-12-23_00000046_1	73,3	70,93	73,78	68,33	72,49	76,22	70,02	70,35
2017-12-23_00000041_1	89,3	89,39	77,63	82,88	70,4	86,37	84,67	87,97
2017-12-23_00000039_1	76,35	72,84	68,1	84,37	76,52	74,26	87,44	63,53
2017-12-23_00000038_1	84,72	88,06	86,33	93,26	90,82	84,2	92,86	72,33
2017-12-16_00000124_1	87,45	77,98	86,1	85,47	59,53	78,36	89,19	84,74
2017-12-16_00000109_1	91,55	75,98	77,41	78,54	62,15	73,73	67,97	55,01
2017-12-16_00000096_1	69,9	84,47	78,45	70,46	79,29	85,78	72,3	81,65
2017-12-16_00000061_1	69,97	69,4	63,87	68,26	63,08	57,11	64,12	64,48
2017-12-16_00000009_1	75,18	86,83	76,95	76,26	70,66	74,88	69,75	78,68
2017-12-16_00000000_1	4,47	46,86	4,32	39,46	15,29	17,95	24,8	24,29
2017-12-09_00000058_1	58,94	69,06	0	0	69,36	71,16	66,27	68,84
2017-12-09_00000057_1	87,14	86,5	66,59	87,38	84,45	87,53	92,07	82,64
2017-12-09_00000050_1	37,76	62,81	32,94	42,41	57,74	50,32	50,85	57,66
2017-12-09_00000037_1	78,28	87,19	90,27	91,39	91,29	88,01	91,8	75,51
2017-12-09_00000018_1	76,95	74,7	80,22	77,01	72,31	75,96	75,19	74,14
2017-12-09_00000015_1	89,57	78,75	88,81	90,62	92,62	79,67	91,64	79,09
2017-12-09_00000004_1	21,74	25,47	21,6	25	27,17	22,73	24,29	21,06
2017-12-02_00000116_1	97,53	71,88	100	100	44,66	83,59	100	48,18
2017-12-02_00000114_1	25,85	26,62	12,24	15,88	22,19	24,94	23,2	28,24
2017-12-02_00000102_1	37,34	68,64	58,55	57,98	69,91	63,58	67,77	67,06

**2017-12-02_00000070_1**	89,5	90,4	93,03	80,64	80,42	68,44	92,59	77,52
**2017-12-02_00000069_1**	73,84	71,88	60,63	68,17	66,75	74,77	63,72	81,83
**2017-12-02_00000058_1**	94,93	87,87	90,61	94,64	93,6	94,01	95,23	88,05
**2017-12-02_00000048_1**	76,25	80,36	76,01	78,07	76,73	76,23	76,5	75,12
**2017-12-02_00000030_1**	93,62	87,72	96,13	87,67	91,6	89,7	90,18	91,97
**2017-12-02_00000023_1**	73,36	72,37	46,12	73,65	68,17	72,79	74,95	71,91
**2017-11-25_00000061_1**	93,88	96,92	89,08	96,98	98,12	89,01	99,26	70,77
**2017-11-25_00000056_1**	81,34	72,09	69,8	68,65	75,31	78,01	78,92	82,28
**2017-11-25_00000043_1**	50,86	51,28	38,13	38,96	51,56	42,31	62,57	63,95
**2017-11-25_00000035_1**	46,8	49,41	14,49	34,97	44,48	51,7	49,9	57,53
**2017-11-25_00000030_1**	75,91	76,1	66,12	68,56	65,77	74,95	62,42	39,67
**2017-11-25_00000022_1**	81,53	78,17	78,99	80,74	78,56	77,5	74,56	81,11
**2017-11-25_00000007_1**	96,02	89,75	92,6	90,8	95,06	86,32	88,9	80,95
**2017-11-18_00000009_1**	99,5	95,68	97,26	96,29	97,17	85,99	94,31	85,63
**2017-11-11_00000077_1**	52,7	37,9	14,56	26,85	29,73	25,61	43,58	38,61
**2017-11-11_00000070_1**	99,25	93,95	95,39	97,72	99,08	91,36	98,07	94,91
**2017-11-11_00000057_1**	91,05	93,54	93,25	93,27	95,7	94,56	93,65	75,46
**2017-11-11_00000052_1**	54,05	60,97	56,81	56,79	59,2	55,64	62,18	58,95
**2017-11-11_00000020_1**	13,25	27,35	13,78	0	19,38	15,22	14,27	17,1
**2017-11-11_00000010_1**	50,63	61,63	52,27	58,65	59,37	62,98	60,99	63,56
**2017-11-11_00000006_1**	91,02	87,93	88,87	91,28	90,4	84,56	92,12	87,36
**2017-11-04_00000068_1**	82,23	89,77	81,5	89,54	76,64	82,66	85,7	86,03
**2017-11-04_00000048_1**	66,33	63,55	62,41	66,39	65,78	59,47	68,1	54,12
**2017-11-04_00000042_1**	81,91	81,57	75,43	74,58	75,53	77,66	76,43	88,33
**2017-11-04_00000032_1**	90,08	86,66	85,71	94,81	77,27	83,7	91,81	81,69
**2017-10-28_00000119_1**	26,57	46	23,37	27,75	31,35	31,43	34,54	30,28
**2017-10-28_00000075_1**	97,6	64,41	97,36	92,95	71,07	89,63	97,06	83,21
**2017-10-28_00000068_1**	60,91	66,59	60,79	62,5	69,48	79,33	76,63	73,53
**2017-10-28_00000061_1**	46,71	58,03	36,22	47,99	51,93	48,55	61,21	59,98
**2017-10-28_00000052_1**	95,82	81,38	94,05	94,9	87,37	77,64	60,54	81,97
**2017-10-28_00000041_1**	81,41	78,58	82,92	84,05	83,27	73,93	84	71,31
**2017-10-21_00000051_1**	80,73	72,95	73,49	68,9	69,87	70,5	75,14	63,23
**2017-10-21_00000034_1**	54,67	48,81	53,99	57,34	49,05	49,32	55,08	51,38
**2017-10-21_00000022_1**	99,5	92,07	89,78	97,95	90,75	91,33	95,67	95,24

**2017-10-21_00000019_1**	96,33	83	92,26	94,3	57,62	86,39	95,87	72,3
**2017-10-21_00000015_1**	93,93	90,25	91,58	92,36	89,69	83,26	91,46	86,96
**2017-10-21_00000006_1**	89,1	86,52	75,81	78,39	85,81	85,82	83,39	86,48

Supplementary Table 5. Raw data for "lDDT BS" column of Table 1. Target ID refers to the CAMEO target.

Raw data – QS-Score		
**Target**	**SWISS-MODEL**	**Robetta**
**2018-01-13_00000068_1**	31,18	29,94
**2018-01-13_00000054_1**	59,37	56
**2018-01-13_00000043_1**	19,47	19,33
**2018-01-13_00000004_1**	48,33	53,84
**2018-01-06_00000155_1**	9,22	0
**2018-01-06_00000150_1**	97,81	96,39
**2018-01-06_00000107_1**	84,98	72,25
**2018-01-06_00000105_1**	94,77	81,89
**2018-01-06_00000025_1**	31,54	26,41
**2018-01-06_00000014_1**	89,21	94,13
**2017-12-30_00000075_1**	75,41	68,74
**2017-12-30_00000014_1**	54,31	51,38
**2017-12-23_00000046_1**	94,9	93,71
**2017-12-16_00000096_1**	30,09	0,19
**2017-12-16_00000058_1**	49,26	48,23
**2017-12-09_00000060_1**	54	50,69
**2017-12-09_00000051_1**	91,84	81,12
**2017-12-09_00000048_1**	71,33	68,2
**2017-12-02_00000102_1**	13,61	24,89
**2017-12-02_00000070_1**	70,21	8,27
**2017-11-18_00000069_1**	74,32	69,04
**2017-11-18_00000025_1**	81,14	72,24
**2017-11-18_00000015_1**	34,46	36,67
**2017-11-18_00000009_1**	93,4	66,25
**2017-11-11_00000052_1**	47,58	68,33
**2017-11-11_00000010_1**	63,99	70,22
**2017-11-04_00000053_1**	89,23	92,03
**2017-11-04_00000042_1**	95,39	75,31
**2017-10-28_00000041_1**	97,51	93,22
**2017-10-21_00000020_1**	86,03	96,53
**2017-10-21_00000015_1**	94,02	91,62
**2017-10-21_00000006_1**	18,54	69,34

Supplementary Table 6. Raw data for "QS-Score" column of Table 1. Target ID refers to the CAMEO target.

Raw data – Model Confidence

Target	SWISS-MODEL	HHpredB	NaiveBLAST	PRIMO	SPARKS-X	RaptorX	IntFOLD4-TS	Robetta
2018-01-13_00000090_1	0,78	0,82	0,62	0,6	0,51	0,59	0,82	0,84
2018-01-13_00000073_1	0,82	0,74	0,56	0,51	0,55	0,52	0,82	0,61
2018-01-13_00000068_1	0,85	0,76	0,52	0,57	0,55	0,56	0,87	0,89
2018-01-13_00000066_2	0,95	0,95	0,75	0,74	0,51	0,88	0,99	0,99
2018-01-13_00000062_1	0,84	0,53	0,51	0,52	0,51	0,68	0,82	0,69
2018-01-13_00000057_1	1	0,77	0,89	0,63	0,53	0,66	0,62	0,98
2018-01-13_00000055_1	0,96	0,84	0,54	0,52	0,56	0,54	0,94	0,98
2018-01-13_00000054_1	0,92	0,5	0,63	0,72	0,57	0,74	0,65	0,93
2018-01-13_00000050_1	0,97	0,64	0,74	0,66	0,51	0,6	0,88	0,96
2018-01-13_00000047_1	-	-	-	-	0,5	0,73	-	0,64
2018-01-13_00000045_2	0,94	0,68	0,71	0,66	0,5	0,57	0,92	0,91
2018-01-13_00000045_1	0,87	0,79	0,72	0,63	0,51	0,65	0,86	0,76
2018-01-13_00000043_1	0,99	0,76	0,78	0,9	0,57	0,52	0,96	1
2018-01-13_00000040_1	0,93	0,58	0,54	0,51	0,5	0,56	0,75	0,66
2018-01-13_00000004_1	0,9	0,79	0,53	0,65	0,52	0,72	0,84	0,89
2018-01-06_00000180_1	0,95	0,87	0,7	0,66	0,5	0,57	0,9	0,5
2018-01-06_00000178_1	0,91	0,91	0,7	0,64	0,5	0,57	0,92	0,5
2018-01-06_00000118_1	0,58	0,96	-	0,59	-	0,64	0,99	0,96
2018-01-06_00000114_1	0,83	0,75	0,52	-	0,5	0,63	0,77	0,72
2018-01-06_00000107_1	0,94	0,72	0,7	0,68	0,5	0,72	0,64	0,77
2018-01-06_00000105_1	0,9	0,76	0,68	0,75	0,5	0,78	0,86	0,72
2017-12-23_00000093_1	0,94	0,74	0,65	0,69	0,51	0,63	0,89	0,88
2017-12-23_00000090_1	0,8	0,58	0,74	0,65	0,5	0,67	0,75	0,71
2017-12-23_00000089_1	0,82	0,83	0,64	0,56	0,75	0,76	0,92	0,61
2017-12-23_00000086_1	0,94	0,65	0,75	0,73	0,51	0,7	0,94	0,87
2017-12-23_00000084_1	0,99	0,96	0,71	0,67	0,53	0,57	0,97	0,95
2017-12-23_00000066_1	0,83	0,73	0,58	0,5	0,5	0,62	0,91	0,8
2017-12-23_00000047_1	0,83	0,66	0,52	0,71	0,52	0,57	0,7	0,64
2017-12-23_00000046_1	0,78	0,72	0,7	0,69	0,59	0,77	0,88	0,83
2017-12-23_00000041_1	0,87	0,73	0,55	0,69	0,54	0,69	0,83	0,86
2017-12-23_00000040_1	0,93	0,91	0,86	0,76	0,52	0,6	0,89	0,91
2017-12-23_00000039_1	0,88	0,74	0,62	0,64	0,51	0,68	0,88	0,85

**2017-12-23_00000038_1**	0,92	0,86	0,71	0,74	0,51	0,75	0,9	0,96
**2017-12-23_00000027_1**	0,82	0,88	-	0,86	0,51	0,64	0,77	0,86
**2017-12-23_00000021_2**	0,91	0,84	0,66	0,65	0,53	0,69	0,92	0,87
**2017-12-23_00000015_1**	0,66	0,91	0,7	-	-	0,67	0,83	0,88
**2017-12-23_00000001_1**	-	0,81	0,64	-	-	0,75	0,77	0,73
**2017-12-16_00000160_1**	0,95	0,75	0,53	0,64	0,5	0,71	0,74	0,81
**2017-12-16_00000124_1**	0,89	0,88	0,56	0,57	0,5	0,65	0,93	0,94
**2017-12-16_00000109_1**	0,9	0,83	0,69	0,68	0,5	0,7	0,86	0,5
**2017-12-16_00000096_1**	0,99	0,81	0,7	0,63	0,51	0,64	0,88	0,88
**2017-12-16_00000083_1**	0,85	0,66	0,55	0,51	0,5	0,54	0,93	0,74
**2017-12-16_00000061_1**	0,88	0,88	0,71	0,71	0,5	0,67	0,91	0,85
**2017-12-16_00000058_1**	0,9	0,91	0,65	0,69	0,51	0,69	0,93	0,85
**2017-12-16_00000045_2**	0,94	1	0,96	0,74	0,75	0,57	0,97	0,99
**2017-12-16_00000044_2**	0,95	0,97	0,89	0,88	0,66	0,64	0,95	0,97
**2017-12-16_00000018_1**	0,87	0,62	0,62	0,64	0,5	0,72	0,92	0,85
**2017-12-16_00000009_1**	0,85	0,7	0,65	0,66	0,5	0,6	0,74	0,74
**2017-12-16_00000002_1**	0,85	0,53	0,65	0,55	0,57	0,66	0,6	0,52
**2017-12-16_00000000_1**	-	0,9	-	-	0,5	0,7	0,86	0,58
**2017-12-09_00000061_1**	0,66	0,78	0,56	0,58	0,52	0,61	0,72	0,69
**2017-12-09_00000060_1**	0,77	0,8	0,54	0,64	0,52	0,63	0,69	0,69
**2017-12-09_00000058_1**	0,83	0,65	0,58	0,51	0,51	0,61	0,7	0,81
**2017-12-09_00000057_1**	0,87	0,86	0,72	0,58	0,6	0,6	0,51	0,87
**2017-12-09_00000051_1**	0,99	0,79	0,55	0,97	0,52	0,68	0,82	0,77
**2017-12-09_00000050_1**	0,92	0,84	0,68	0,54	0,5	0,75	0,89	0,93
**2017-12-09_00000048_1**	0,67	0,75	0,71	0,65	0,54	0,58	0,53	0,89
**2017-12-09_00000046_1**	0,83	0,8	0,6	-	0,51	0,63	0,77	0,69
**2017-12-09_00000040_1**	0,51	0,96	0,55	-	0,5	0,59	0,99	0,97
**2017-12-09_00000037_1**	0,99	0,8	0,93	0,85	0,54	0,58	1	0,94
**2017-12-09_00000021_1**	0,76	0,65	-	-	0,54	0,71	0,89	0,71
**2017-12-09_00000018_1**	0,96	0,83	0,61	0,8	0,51	0,63	0,87	0,83
**2017-12-09_00000015_1**	0,99	0,78	0,84	0,88	0,5	0,75	0,99	0,97
**2017-12-09_00000010_2**	0,98	0,81	0,72	0,82	0,5	0,89	0,92	0,91
**2017-12-09_00000008_2**	0,94	0,9	0,61	0,64	0,54	0,61	0,91	0,94
**2017-12-09_00000005_1**	0,85	0,74	0,82	0,53	0,56	0,5	0,74	0,95
**2017-12-09_00000004_1**	0,76	0,98	0,53	0,51	0,51	0,57	0,96	0,95

**2017-12-02_00000122_1**	1	0,97	0,53	0,58	0,52	0,56	0,99	1
**2017-12-02_00000116_1**	0,95	0,91	0,74	0,61	0,57	0,63	1	0,89
**2017-12-02_00000114_1**	0,89	0,88	0,58	0,66	0,5	0,69	0,92	0,54
**2017-12-02_00000109_1**	0,79	0,61	0,68	0,56	0,72	0,65	0,74	0,66
**2017-12-02_00000106_1**	0,93	0,85	0,88	0,82	0,52	0,67	0,68	0,85
**2017-12-02_00000102_1**	0,82	0,8	0,57	0,72	0,52	0,73	0,89	0,67
**2017-12-02_00000078_1**	0,82	0,8	-	0,72	0,55	0,63	0,88	0,85
**2017-12-02_00000076_1**	0,84	0,78	0,79	0,64	0,54	0,61	0,88	0,88
**2017-12-02_00000071_1**	0,93	0,84	0,89	0,69	0,52	0,73	0,88	0,9
**2017-12-02_00000070_1**	0,96	0,84	0,73	0,77	0,5	0,66	0,75	0,74
**2017-12-02_00000069_1**	0,79	0,64	0,63	0,56	0,51	0,56	0,73	0,78
**2017-12-02_00000058_1**	0,85	0,79	0,75	0,67	0,53	0,52	0,85	0,99
**2017-12-02_00000048_1**	0,81	0,67	0,59	0,67	0,5	0,63	0,76	0,71
**2017-12-02_00000042_2**	0,73	0,62	0,72	0,67	0,5	0,61	0,69	0,66
**2017-12-02_00000042_1**	0,89	0,74	0,61	0,72	0,53	0,62	0,91	0,88
**2017-12-02_00000030_1**	0,88	0,76	0,91	0,6	0,5	0,62	0,85	1
**2017-12-02_00000023_1**	0,84	0,76	0,59	0,68	0,5	0,66	0,82	0,84
**2017-11-25_00000069_1**	0,83	0,64	0,67	0,64	0,5	0,75	0,8	0,78
**2017-11-25_00000066_1**	0,85	0,66	0,77	-	0,5	0,69	0,83	0,85
**2017-11-25_00000061_1**	0,95	0,84	0,69	0,71	0,52	0,69	0,91	0,87
**2017-11-25_00000060_1**	0,9	0,88	0,67	0,75	0,52	0,64	0,96	0,94
**2017-11-25_00000056_1**	0,93	0,87	0,67	0,69	0,52	0,69	0,88	0,91
**2017-11-25_00000055_1**	-	0,8	0,95	0,69	0,5	0,56	0,61	0,89
**2017-11-25_00000050_1**	0,88	0,82	0,7	0,65	0,5	0,69	0,87	0,83
**2017-11-25_00000043_1**	0,95	0,55	0,65	0,63	0,51	0,69	0,82	0,66
**2017-11-25_00000041_1**	0,82	0,61	0,55	0,57	0,5	0,59	0,81	0,76
**2017-11-25_00000035_1**	0,89	0,85	0,73	0,78	0,5	0,66	0,86	0,89
**2017-11-25_00000030_1**	0,76	0,71	0,61	0,62	0,5	0,67	0,68	0,75
**2017-11-25_00000022_1**	0,94	0,83	0,67	0,67	0,5	0,64	0,83	0,94
**2017-11-25_00000021_1**	0,5	0,64	0,6	0,55	0,5	0,58	0,68	0,58
**2017-11-25_00000020_1**	0,93	0,63	0,78	0,69	0,51	0,74	0,74	0,7
**2017-11-25_00000018_1**	0,97	0,61	0,72	0,73	0,5	0,66	0,68	0,97
**2017-11-25_00000017_1**	0,72	0,73	0,62	0,65	0,64	0,5	0,88	0,84
**2017-11-25_00000007_1**	0,86	0,76	0,72	0,67	0,5	0,65	0,78	0,86
**2017-11-18_00000073_1**	0,95	0,9	0,69	0,64	0,52	0,58	0,96	0,93

**2017-11-18_00000069_1**	1	0,67	0,68	0,92	0,53	0,65	-	0,67
**2017-11-18_00000068_1**	0,84	0,83	0,62	0,62	0,55	0,62	0,95	0,91
**2017-11-18_00000014_1**	0,95	0,64	0,6	0,66	0,5	0,71	0,65	0,57
**2017-11-18_00000009_1**	0,87	0,8	0,61	0,59	0,56	0,56	0,85	0,6
**2017-11-11_00000093_1**	-	-	-	-	-	-	-	0,54
**2017-11-11_00000092_1**	0,81	1	0,95	0,7	0,5	0,62	1	0,79
**2017-11-11_00000086_1**	0,74	0,71	0,58	0,52	0,5	0,63	0,85	0,75
**2017-11-11_00000077_1**	0,91	0,86	-	-	0,5	0,69	0,78	0,91
**2017-11-11_00000070_1**	0,68	1	0,78	0,72	0,5	0,57	0,99	0,54
**2017-11-11_00000057_1**	0,82	0,86	0,62	0,69	0,5	0,61	0,85	0,5
**2017-11-11_00000054_1**	0,82	0,79	0,66	0,6	0,51	0,61	0,64	0,62
**2017-11-11_00000052_1**	0,79	0,78	0,68	0,69	0,5	0,69	0,81	0,78
**2017-11-11_00000036_1**	0,78	0,64	0,5	0,52	0,58	0,63	0,81	0,78
**2017-11-11_00000027_2**	0,99	0,91	0,83	0,65	0,55	0,68	0,98	0,92
**2017-11-11_00000026_2**	0,96	0,93	0,85	0,82	0,52	0,69	0,95	0,93
**2017-11-11_00000025_2**	0,96	0,93	0,82	0,73	0,52	0,72	0,97	0,94
**2017-11-11_00000024_2**	0,93	0,81	0,66	0,74	0,52	0,71	0,84	0,85
**2017-11-11_00000020_1**	0,63	0,83	0,6	0,6	0,51	0,58	0,74	0,66
**2017-11-11_00000016_2**	0,69	0,89	0,59	0,7	0,68	1	0,96	0,88
**2017-11-11_00000010_1**	0,84	0,8	0,58	0,65	0,51	0,61	0,8	0,86
**2017-11-11_00000006_1**	0,69	0,52	0,67	0,78	0,5	0,82	0,64	0,75
**2017-11-04_00000068_1**	0,97	0,73	0,71	0,77	0,5	0,66	0,98	0,89
**2017-11-04_00000055_2**	0,95	0,95	0,75	0,67	0,5	0,76	0,98	0,91
**2017-11-04_00000048_1**	0,86	0,65	0,63	0,51	0,55	0,59	0,85	0,77
**2017-11-04_00000042_1**	0,9	0,81	0,69	0,69	0,52	0,77	0,86	0,92
**2017-11-04_00000037_1**	0,94	0,83	0,69	0,68	0,5	0,65	0,89	0,87
**2017-11-04_00000032_1**	0,88	0,58	0,59	0,74	0,56	0,58	0,81	0,85
**2017-11-04_00000023_2**	0,81	0,87	0,71	0,83	0,5	0,68	0,67	0,76
**2017-11-04_00000020_2**	0,85	0,88	0,65	0,61	0,5	0,51	0,78	0,82
**2017-11-04_00000013_1**	0,87	0,83	0,74	0,75	0,5	0,64	0,85	0,88
**2017-11-04_00000009_1**	0,8	0,8	0,54	-	-	0,55	0,85	0,64
**2017-10-28_00000134_1**	0,77	0,87	0,59	0,57	0,75	0,51	0,9	0,98
**2017-10-28_00000126_1**	0,89	0,82	0,72	0,75	0,51	0,62	0,89	0,96
**2017-10-28_00000119_1**	0,79	0,86	0,71	0,63	0,5	0,65	0,95	0,9
**2017-10-28_00000109_1**	0,68	0,58	0,62	0,52	0,5	0,54	0,85	0,85

**2017-10-28_00000099_2**	0,96	1	-	-	0,5	0,8	-	0,5
**2017-10-28_00000075_1**	0,84	0,71	0,63	0,76	0,51	0,61	0,83	0,87
**2017-10-28_00000074_1**	0,86	0,85	-	-	0,5	0,67	0,92	0,92
**2017-10-28_00000068_1**	0,69	0,73	0,67	0,6	0,53	0,67	0,72	0,72
**2017-10-28_00000067_1**	0,75	0,83	0,68	0,64	0,51	0,69	0,87	0,99
**2017-10-28_00000062_1**	0,72	0,71	0,66	0,67	0,53	0,58	0,94	0,69
**2017-10-28_00000061_1**	0,55	0,57	0,63	0,62	0,51	0,59	0,87	0,92
**2017-10-28_00000052_1**	0,82	0,87	0,63	0,6	0,51	0,55	0,83	0,92
**2017-10-28_00000044_1**	0,93	0,84	0,51	0,57	0,52	0,74	0,71	0,96
**2017-10-28_00000041_1**	-	0,56	0,68	0,91	0,82	0,68	0,91	0,86
**2017-10-28_00000029_1**	0,73	0,84	0,74	0,57	0,56	0,77	0,95	0,92
**2017-10-28_00000027_2**	0,91	0,91	0,63	0,68	0,5	0,64	0,95	0,91
**2017-10-28_00000021_1**	0,9	0,81	0,67	0,68	0,5	0,66	0,87	0,91
**2017-10-21_00000060_2**	-	0,95	-	-	-	-	-	0,59
**2017-10-21_00000059_1**	0,91	0,71	-	0,76	0,51	0,68	0,91	0,51
**2017-10-21_00000051_1**	0,79	0,91	0,66	0,7	0,52	0,62	0,96	0,91
**2017-10-21_00000036_1**	0,7	0,84	0,67	0,73	0,62	0,68	0,86	0,96
**2017-10-21_00000034_1**	0,79	0,81	0,63	0,73	0,68	0,51	0,94	0,98
**2017-10-21_00000032_1**	0,79	0,79	0,75	0,64	0,85	0,72	0,87	0,91
**2017-10-21_00000031_1**	0,83	0,98	0,55	0,89	0,71	0,74	0,77	0,86
**2017-10-21_00000029_1**	0,64	0,78	0,79	0,69	0,69	0,6	0,88	0,74
**2017-10-21_00000026_1**	0,59	0,69	0,72	0,67	0,53	0,69	0,63	0,5
**2017-10-21_00000023_1**	0,64	0,54	0,69	0,71	0,51	0,6	0,76	0,57
**2017-10-21_00000022_1**	-	0,85	1	-	0,62	0,75	1	1
**2017-10-21_00000020_1**	-	-	0,79	0,67	-	0,73	-	-
**2017-10-21_00000019_1**	0,84	0,85	0,7	0,69	0,5	0,67	0,86	0,91
**2017-10-21_00000015_1**	0,94	0,89	0,72	0,76	0,51	0,75	0,91	0,88
**2017-10-21_00000012_1**	0,92	0,86	-	0,99	0,52	0,61	0,91	0,63
**2017-10-21_00000006_1**	0,91	0,71	0,56	0,57	0,51	0,62	0,89	0,93
**2017-10-21_00000005_1**	0,9	0,89	0,78	0,7	0,5	0,61	0,85	0,9
**2017-10-21_00000002_1**	0,71	0,74	0,89	0,64	0,76	0,63	0,86	1

Supplementary Table 7. Raw data for "Model Confidence" column of Table 1. Target ID refers to the CAMEO target. Please note that the model confidence is not defined in CAMEO when the local IDDT values for a given model are all greater or smaller than 0.6.
